# Identification of microRNAs implicated in the late differentiation stages of normal B cells suggests a central role for miRNA targets ZEB1 and TP53

**DOI:** 10.18632/oncotarget.14683

**Published:** 2017-01-17

**Authors:** Giorgio Malpeli, Stefano Barbi, Simonetta Zupo, Gabriele Tosadori, Giovanni Scardoni, Anna Bertolaso, Silvia Sartoris, Stefano Ugel, Caterina Vicentini, Matteo Fassan, Annalisa Adamo, Mauro Krampera, Maria Teresa Scupoli, Carlo Maria Croce, Aldo Scarpa

**Affiliations:** ^1^ Department of Surgical Sciences, Dentistry, Gynecology and Pediatrics, Section of Surgery, University of Verona, Verona, Italy; ^2^ Department of Diagnostics and Public Health, Section of Pathological Anatomy, University of Verona, Verona, Italy; ^3^ Laboratory of Molecular Diagnostics, IRCCS-AOU San Martino-IST, Istituto Nazionale per la Ricerca sul Cancro, Genoa, Italy; ^4^ Center for BioMedical Computing (CBMC), University of Verona, Verona, Italy; ^5^ Department of Medicine, Section of Immunology, University of Verona, Verona, Italy; ^6^ Department of Medicine, Surgical Pathology and Cytopathology Unit, University of Padua, Padua, Italy; ^7^ Department of Medicine, Section of Hematology, Stem Cell Research Laboratory, University of Verona, Italy; ^8^ Department of Medicine, Section of Hematology, University of Verona, Italy; ^9^ Department of Molecular Virology, Immunology and Medical Genetics, Comprehensive Cancer Center, The Ohio State University, Columbus, OH, USA; ^10^ Applied Research on Cancer-Network (ARC-NET), University of Verona, Verona, Italy

**Keywords:** B cell development, follicle, germinal centre, microRNAs, network analysis

## Abstract

In the late B cell differentiation stages, miRNAs expression modifications promoting or inhibiting key pathways are only partially defined. We isolated 29 CD19^+^ human B cell samples at different stages of differentiation: B cells from peripheral blood; naïve, germinal center (GC) and subepithelial (SE) B cells from tonsils. SE cells were further split in activated and resting B cell. The miRNA expression profile of these B cells was assessed by microarray analysis and selected miRNAs were validated by quantitative RT-PCR and *in situ* hybridization on normal tonsils. The comparison of all samples showed changes in 107 miRNAs in total. Among 48 miRNAs differentially expressed in naïve, GC and SE cells, we identified 8 miRNAs: mir-323, mir-138, mir-9*, mir-211, mir-149, mir-373, mir-135a and *mir-184*, strictly specific to follicular cells that had never been implicated before in late stages of B cell development. Moreover, we unveiled 34 miRNAs able to discriminate between CD5^−^ activated B cells and resting B cells. The miRNAs profile of CD5^−^ resting B cells showed a higher similarity to naïve CD5^+^ than CD5^−^ activated B cells. Finally, network analysis on shortest paths connecting gene targets suggested *ZEB1* and *TP53* as key miRNA targets during the follicular differentiation pathway. These data confirm and extend our knowledge on the miRNAs-related regulatory pathways involved in the late B cell maturation.

## INTRODUCTION

Newly formed immature B cells, that express an unrefined antigen receptor, are released from the bone marrow to blood circulation and they conclude their differentiation in the follicles of peripheral lymphoid organs [1]. In the immune follicles, a combination of death and survival stimuli results in T-cell dependent selection of functionally mature IgV class-switched and hypermutated antibody-producing B lymphocytes [2]. Ontogenesis of B cells is accomplished under the concerted action of multiple factors performing transcriptional programs entailing down- or up- modulation of genes at each stage of maturation [3, 4]. In this scenario, MicroRNAs (miRNAs) play their own part as well [5].

MiRNAs are non-protein coding RNAs active in the regulation of gene transcription and mRNA translation. MiRNAs action in concert with transcriptional inhibitors and enhancers results in the fine tuning of gene expression. As a consequence, miRNAs influence many cellular processes, such as cell identity, growth, proliferation and control of cell death [6].

Multiple evidences indicated a critical role of specific miRNAs in B cell development [7–9]. In the follicles, during late maturation stages, the down-regulation of *mir-150*, which controls the transcription factor c-Myb, is requested for the germinal centre (GC) selection and for a correct development of the adaptive humoral immune response [10]. Other miRNAs such as *mir-155*, *mir-181b*, *mir-15a*, *mir-16*, *mir-15b*, *mir-34a*, *mir-9*, *mir-30*, *let-7a*, *mir-125b*, *mir-217* and *mir-185* modulate the expression of pivotal genes and functions which contribute to the final B-cell maturation [6]. Also the couple *mir-150*/c-Myb is involved in the B cell lineage differentiation pathway [11].

The GC is a transient structure that forms within peripheral lymphoid organs in response to B lymphocyte stimulation by T cell-dependent antigens, with the involvement of other immune cells. Up- and down-regulation of multiple miRNAs was detected in B cells at sequential stages of maturation during the GC reaction [12–14]. Some of the miRNAs highlighted by profiling studies were then demonstrated to be functionally relevant [11]. Thus, it appears crucial to identify selective miRNAs that influence and drive B cell development to better characterize the molecular pathways involved in this biological process.

The marginal zone B cells population is heterogeneous, comprising naïve and memory cells [15, 16]. The subepithelial region of tonsils, corresponding to the marginal zone of other peripheral lymphoid organs, on the basis of the pattern of surface markers, contains two subpopulations of supposedly mature CD5^−^ B cells: resting and activated [17]. Activated B cells are IgV gene-hypermutated B cells that achieved the final maturation. Instead, similarly to mice, human SE resting B cells could represent a subpopulation of IgV gene-unmutated B cells survived to the GC selection and waiting for activation by a specific antigene to complete their maturation process [18, 19]. As the miRNAs expression pattern characterizes different B cell maturation stages, it could also contribute to define the ontogenesis of these two B cell subsets.

In this study, we compared the expression profiles of miRNAs in CD19^+^ B cells at different late differentiation steps by microarray analysis. In addition, we compared the expression profile of miRNAs in subepithelial activated and resting B cells, to better define the identity of the two B cell subtypes. Experimentally validated gene targets of differentially expressed miRNAs were subjected to network analysis, to infer the underlying regulatory pathways. By this procedure, we tried to identify protein hubs and cell functions operated by miRNAs.

## RESULTS

### One hundred and seven single miRNAs were significantly differentially expressed in CD19^+^ B cells from blood compared to B cells from tonsils at different stages of activation

We assessed miRNAs expression in B cell subsets representing stages of development ranging from CD19^+^ peripheral blood B cells to mature B cells in the immune follicles of tonsils. We analyzed a total of 29 B cell samples by microarray technology: four samples of CD19^+^ B cells isolated from peripheral blood; 14 B cell samples isolated from tonsil follicles, that were divided in three different groups: naïve CD5^+^ B cells (2 samples), CD23^−^CD39^−^ GC B cells (12 samples) and CD5^−^ SE mature B cells (11 samples). Finally, among the 11 samples of mature CD5^−^ B cells, we defined two distinctive subgroups: the IgV-hypermutated activated B cells (4 samples) and the IgV-non-hypermutated resting B-cells (seven samples). On summary, on the basis of the differentiation stage, our B cell samples could be divided in four different experimental groups, including the subepithelial CD5^−^ B cells which are subdivided in two subgroups.

The heat map in Supplementary Figure 1 shows the levels of differentially expressed miRNAs among four B-cell subsets. The hierarchical clustering emphasized the correlation among samples belonging to the same differentiation stage (Supplementary Figure 1A). One hundred and seven single miRNAs were differentially expressed among the five B-cell subsets at FDR 1% (Supplementary Figure 1B). The list of 135 probes representing 107 single miRNAs is reported in Supplementary Table 1.

Most of miRNA expression differences occurred between CD19^+^ and naïve CD5^+^ cells. Among follicular B cell populations, CD23^−^CD39^−^ GC B-cells showed an expression profile well distinct from that of naïve CD5^+^ B cells and mature CD5^−^ B cells. Indeed, these latter two cell populations showed only a limited number of differentially expressed miRNAs. Therefore, this analysis highlighted the strong modulation of miRNA expression during the late B cell differentiation.

### The expression of thirty-seven single miRNAs distinguishes peripheral blood CD19^+^ from CD19^+^/CD5^+^ B cells of lymph node

About 70% of peripheral blood CD19^+^ cells are immature or naïve CD19^+^/CD5^−/+^ B lymphocytes before joining peripheral lymphoid compartments for the achievement of the antigen-dependent final maturation as high affinity antibody secreting plasma cells [20]. When these immature B cells reach the microenvironment of the white zone of the immune follicles, they become CD5^+^ B cells. To identify miRNAs involved in this B cell transition, we compared the miRNA profile of CD19^+^ B cells isolated from blood to the miRNA profile of CD5^+^ B cells isolated from tonsils: the expression levels of 37 miRNAs appeared changed in a statistically significant manner (FDR 5%). Among these differently expressed miRNAs, 22 were downregulated, whilst, on the contrary, 15 miRNAs were upregulated in CD5^+^ B cells compared to CD19^+^ B cells (Supplementary Figure 2, Supplementary Table 2). Downregulated miRNAs in CD5^+^ cells included members of both the *mir-29* and *mir-30* families as well as the *mir-17-92* cluster. On the contrary, we identified, among the upregulated miRNAs, members of the *mir-132* and *mir-212* families, that generated a specific cluster on chromosome 17.

### Modulation of miRNAs expression allows the clusterization of naïve, GC and mature SE B cells samples

**Figure 1:**
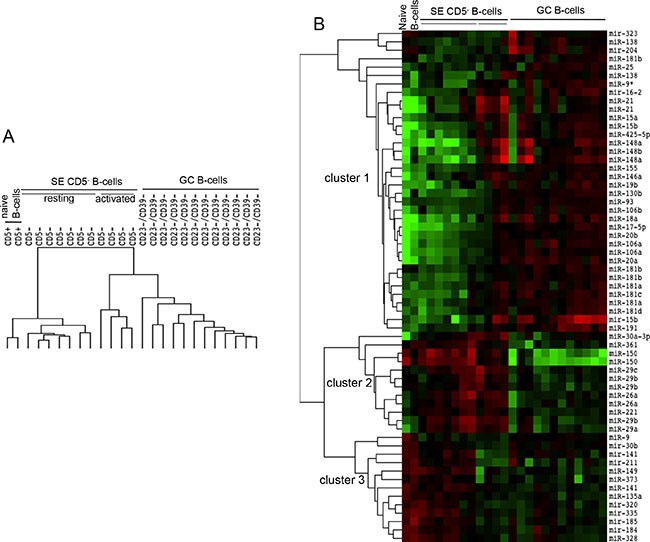
Expression profile of miRNAs in cell subsets representing different stages of B cell maturation CD5^+^: naïve B cells from tonsils; CD23^−^CD39^−^: germinal centre (GC) B cells from tonsils; CD5−: subepithelial (SE) mature B cells from tonsils, subdivided in CD5^−^ resting and CD5− activated B cells. **(A)** Array tree of 25 samples representing different stages of maturation of the B cells based on the expression levels of miRNAs. **(B)** The heat map describes the expression levels of 48 differentially expressed miRNAs in 25 samples owning to four B cell subsets isolated from tonsils (FDR 1%). Clusters 1, 2 and 3 are three miRNAs groups isolated by clustering procedures. Red, higher expression (log2, +4); green, lower expression (log2, −4).

**Figure 2:**
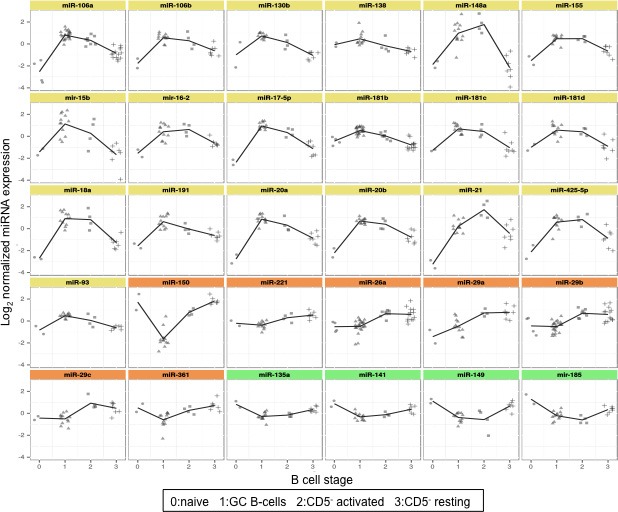
Expression levels of top 30 differentially expressed miRNAs in cell subsets representing different stages of late B cell differentiation Graphs report expression levels of 30 miRNAs with lower Q value among the 48 differentially expressed miRNAs in 29 samples divided in four B cell subsets. Color graphs indicate the miRNAs included in Cluster 1 (yellow), Cluster 2 (orange) and Cluster 3 (green). Data were obtained from microrray analysis after normalization and log2 transformation of the probe signal. 0: naïve B cells; 1: germinal centre B cells; 2: subepithelial activated B cells; 3: subepithelial resting B cells.

To identify miRNAs that are actively modulated during the GC maturation, we compared the expression profiles of miRNAs obtained from three main follicular B cell populations: naïve B cells (CD5^+^), GC B cells (CD23^−^CD39^−^) and mature SE B cells (CD5^−^). Statistical procedures clustered three homogeneous groups of samples (Figure 1A). Moreover, CD5^−^ B cell samples were split in the two different clusters of activated and resting. Forty-eight single miRNAs, corresponding to 61 spots, were significantly differentially expressed among the 25 samples (at FDR 1%) and they were clusterized in three main groups: cluster 1, composed by 28 miRNAs; cluster 2, composed by 8 miRNAs; and cluster 3 composed by 12 miRNAs (Figure 1B). Cluster 1 included miRNAs whose expression increased in the passage from naïve B cells to GC B-cells and activated CD5^−^ B cells. Moreover, *miR-323*, *mir-138* and *miR-204* were more highly expressed in naïve and SE B cells. Cluster 2 comprised miRNAs downregulated in GC B cells compared to naïve and CD5^−^ activated B cells. Finally, cluster 3 included miRNAs whose expression decreased during the transition from CD5^+^ to CD23^−^CD39^−^ and activated CD5^−^ B cells (Figure 2). Considering all differentially expressed miRNAs, we detected *miR-150*, *miR-361*, *miR-130b*, *miR-181b* and members of miRNA clusters *miR-17-5p*, *miR-106a*, *miR-20a* and *miR-20b* as the most variable miRNAs (FDR = 0.0077) (Table 1).

**Table 1:** List of differentially expressed miRNAs among CD5^+^ B cells, CD23^−^/CD39^−^ B cells and CD5^−^ B cells (FDR 2%)

miRNA	*Q* value
*miR-150*	0.0077
*miR-17-5p*	0.0077
*miR-106a*	0.0077
*miR-20a*	0.0077
*miR-181b*	0.0077
*miR-361*	0.0077
*miR-130b*	0.0077
*miR-20b*	0.0077
*miR-18a*	0.0081
*miR-29b*	0.0082
*miR-148a*	0.0082
*miR-191*	0.0083
*miR-93*	0.0086
*miR-26a*	0.0086
*miR-221*	0.0086
*miR-155*	0.0087
*mir-15b*	0.0087
*miR-29a*	0.0087
*miR-138*	0.0098
*miR-106b*	0.0102
*miR-135a*	0.0102
*miR-21*	0.0102
*mir-16-2*	0.0102
*miR-181c*	0.0102
*miR-141*	0.0102
*mir-185*	0.0102
*miR-149*	0.0106
*miR-181d*	0.0110
*miR-425-5p*	0.0110
*miR-29c*	0.0110
*miR-181a*	0.0110
*miR-25*	0.0124
*mir-204*	0.0136
*mir-184*	0.0136
*miR-148b*	0.0136
*miR-373*	0.0138
miR-9*	0.0138
*mir-30b*	0.0138
*miR-19b*	0.0147
*mir-320*	0.0153
*miR-146a*	0.0153
*miR-30a-3p*	0.0156
*miR-9*	0.0156
*mir-323*	0.0159
*mir-211*	0.0159
*miR-328*	0.0164
*mir-335*	0.0178
*miR-15a*	0.0198

MiRNAs belonging to the cluster *mir-17/92* and the paralogous clusters *mir-25/106b* and *mir-106a/363* showed a similar trend of expression, i.e. *mir-17-5p*, *mir-20a*, *mir-106a*, *mir-20b*, *mir-18a*, *mir-106a*, *mir-18b*, *mir-20b*, *mir-106b*, *mir-93* and *mir-25* (Cluster 1, Figure 1). The same expression pattern was also present in the cluster of *mir-191/425*, (Cluster 1, Figure 1).

The average expression levels of the top 20 significantly differentially expressed miRNAs (Figure 2) demonstrated that they were mostly upregulated in mature B cells. In contrast, *miR-150*, *miR-361*, *miR-221*, *miR-135a*, *miR-141*, *miR-185* and *miR-149* decreased in GC B cells compared to naïve B cells.

Finally, naïve CD5^+^ B-cells shared with activated CD5^−^ B-cells a specific group of miRNAs whose expression resulted downregulated in CD23^−^CD39^−^ B-cells (Figure 1). In addition, among miRNAs expressed at higher level in CD5^−^ B cells compared to CD5^+^ B cells, we identified five miRNAs: *miR-29a*, *miR-29b*, *miR-29c*, *miR-26a* and *miR-221*. The expression level of these miRNAs was comparable to those of CD23^−^CD39^−^ B cells. Therefore, we can conclude that the B cell maturation step allowing the entrance in peripheral lymphoid organs induces the upregulation of a restricted group of miRNAs: *miR-29a*, *miR-29b*, *miR-29c*, *miR-26a* and *miR-221*.

To better reinforce our original data, by comparing our results with published data we generated a list of 48 differentially expressed miRNAs [12–14] (Table 2). The four studies considered for the comparison, including the present study, demonstrated the higher expression in naïve B-cells of *mir-320*, the up-regulation of *mir-181b*, *mir-25*, *miR-130b* in GC B cells as well as the greater expression of both *mir-*29a and seven members linked to the cluster *miR-17/92* in mature B cells. Moreover, in at least one of the four studies, 35 of 48 differentially expressed miRNAs were expressed at higher level in different B cell subsets; on the contrary, 27 miRNAs were not differentially expressed or not detected. However the four studies presented a controversial expression of *mir-185*: we detected levels of *miR-185* higher in naïve than in GC-restricted B cells (Figure 1), whilst both Malumbres et al. [12] and Belver et al. [21] showed *miR-185* upregulation in GC B cells.

**Table 2:** B cell subsets with highest level of miRNAs significantly modulated during the late differention of B cells: a comparison with literature data

miRNA	Malpeli G et al.	Zhang J et al.	Malubres S et al.	Tan LP et al.
*miR-323*	GC	ND	ND	ND
*miR-138*	GC	ND	ND	ND
*miR-204*	GC	GC	ND	ND
*miR-181b*	GC	GC	GC	GC
*miR-25*	GC	GC	GC	GC
miR-9*	GC	ND	ND	ND
*miR-16-2*	GC	ND	GC	GC
*miR-15a*	GC	GC	ND	ND
*miR-15b*	GC	GC	GC	ND
*miR-425-5p*	GC	ND	ND	GC
*miR-148a*	GC	Mat	GC	GC
*miR-148b*	GC	ND	GC	Naïve
*miR-155*	GC	ND	GC	ND
*miR-146a*	GC	GC-Mat	Mat	ND
*miR-19b*	GC	GC	GC	GC
*miR-130b*	GC	GC	GC	GC
*miR-93*	GC	GC	GC	GC
*miR-106b*	GC	GC	GC	GC
*miR-18a*	GC	GC	GC	GC
*miR-17-5p*	GC	GC	GC	GC
*miR-20b*	GC	GC	GC	GC
*miR-106a*	GC	GC	GC	GC
*miR-20a*	GC	GC	GC	GC
*miR-181a*	GC	ND	GC	GC
*miR-181c*	GC	ND	Naïve-GC	ND
*miR-181d*	GC	GC	Naïve-GC	ND
*miR-191*	GC	ND	GC	GC
*miR-21*	Mem	Mem	GC	Mem
*miR-30a-3p*	Mem	GC	GC	ND
*miR-361*	Mem	Mem	ND	GC
*miR-29c*	Mem	Mem-Naïve	GC	Mem
*miR-29b*	Mem	ND	GC	Mem-Naïve
*miR-29a*	Mem	Mem-Naïve	Mem	Mem
*miR-26a*	Mem	Naïve	Naïve	Mem
*miR-221*	Mem	Mem	Naïve	Naïve-Mem
*miR-150*	Naïve	ND	Mem	Naïve
*miR-9*	Naïve	GC	ND	ND
*miR-30b*	Naïve	GC	Mem	GC
*miR-141*	Naïve	Naïve	ND	ND
*miR-211*	Naïve	ND	ND	ND
*miR-149*	Naïve	ND	ND	ND
*miR-373*	Naïve	ND	ND	ND
*miR-135a*	Naïve	ND	ND	ND
*miR-320*	Naïve	Naïve-Mem	Naïve-Mem	Naïve-Mem
*miR-335*	Naïve	Naïve	ND	ND
*miR-185*	Naïve	ND	GC	ND
*miR-184*	Naïve	ND	ND	ND
*miR-328*	Naïve	ND	GC-Mem	ND

ND: not detected or not differentially expressed; Naïve: naïve B cells; GC: germinal centre B cells; Mat: mature B cells. Zhang J et al. [14], Malubres S et al. [12], Tan LP et al. [13].

Our study identified 8 new differentially expressed miRNAs: *mir-323*, *mir-138*, mir-9*, *mir-211*, *mir-149*, *mir-373*, *mir-135a* and *mir-184*; that have not been reported in literature so far.

### Expression of thirty-three selective miRNAs discriminates subepithelial resting and marginal zone CD5^−^ activated B-cells

**Figure 3:**
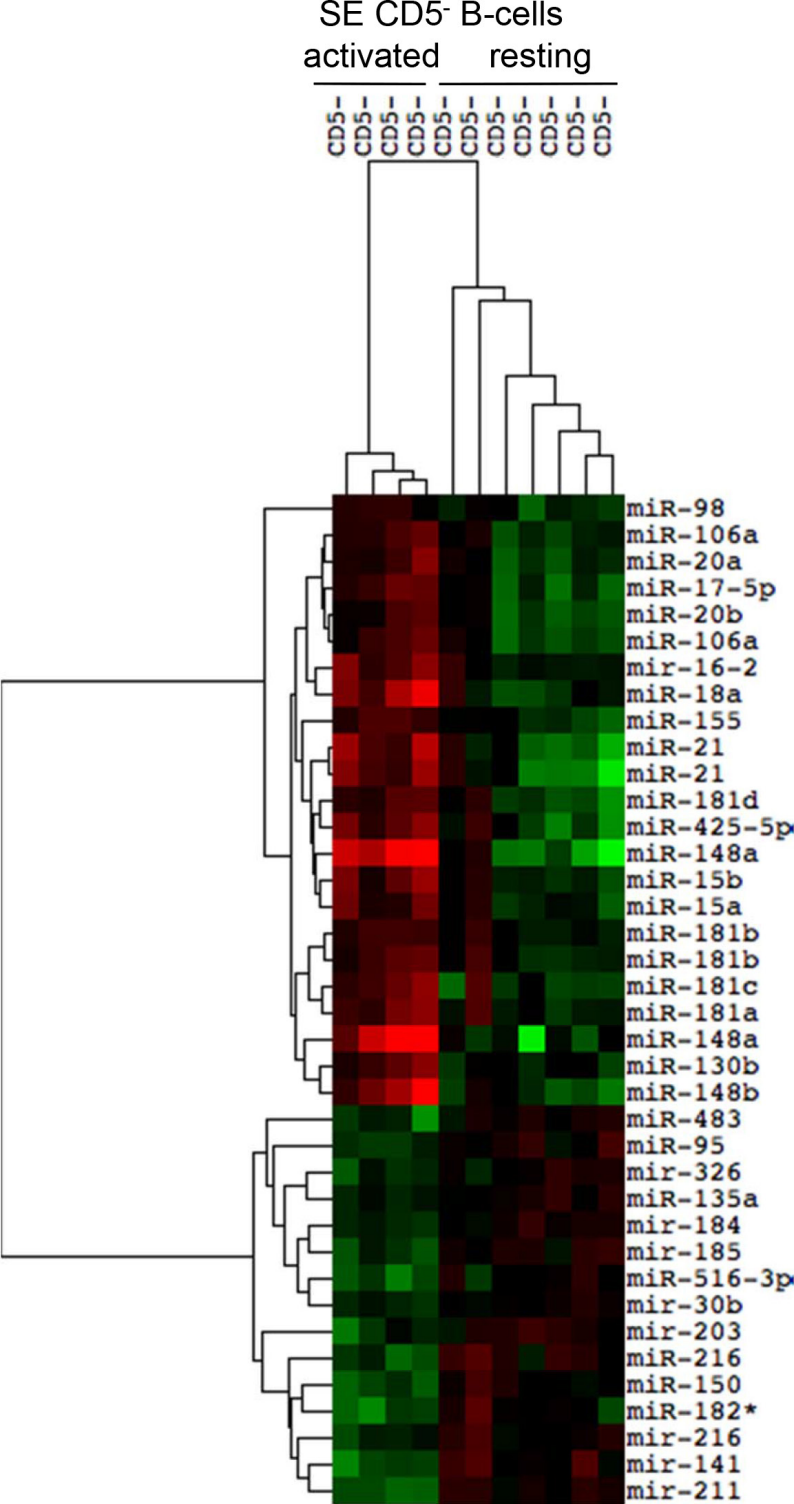
Differential expression of miRNAs in subepithelial CD5− activated and resting B cell subsets The heat map reports the expression levels of differentially expressed miRNAs between two subepithelial (SE) CD5− B cell populations (FDR 10%): activated IgV mutated SE B cells and resting IgV unmutated SE B cells. Red, higher expression (log2, +4); green, lower expression (log2, −4).

As described by Dono M et al., 2000 [17], resting CD5^−^ B cells were identified in the SE region of tonsils as small size CD5^−^ B cells expressing unmutated IgV genes. The ontogenesis of this cell population has not been defined yet, as well as the difference between resting and activated B cells, generated from B cells of GCs adjacent to SE. Therefore, we proceeded to analyze the miRNAs profiles of these two B cell subtypes, comparing four samples of CD5^−^ activated B cells to seven samples of CD5^−^ resting B cells (Figure 3). The two cell populations clustered separately and they are strongly distinguishable for the differential expression of 34 miRNAs (FDR 5%). The miRNAs profile comparison between resting and activated B cells showed the up-regulation of 19 miRNA in activated B cells: *mir-98, mir-106a, mir-20a, mir-17-5p, mir-20b, mir-16-2, mir-18a, mir-155, mir-21, mir-181d, mir-425-5p, mir-148a, mir-15b, mir-15a, mir-181b mir-181c, mir-181a*, *mir-130b*, *mir-148b* (Table 3). Conversely, 15 miRNAs resulted downregulated in activated B cells: mir-483, mir-95, mir-326, mir-135a, mir-184, mir-185, mir-516-3p, mir-30b, mir-203, mir-216, mir-150, mir-182*, mir-141 and mir-211 (Table 3).

**Table 3:** List of differentially expressed miRNAs between subepithelial CD5^−^ activated and resting B cells (FDR 10%)

miRNA	*activated vs resting	*Q* value
*mir-211*	Down	0.0026
*miR-148a*	Up	0.0340
*mir-141*	Down	0.0445
*miR-155*	Up	0.0445
*mir-184*	Down	0.0445
*miR-15b*	Up	0.0445
*mir-30b*	Down	0.0445
*miR-150*	Down	0.0445
*miR-148b*	Up	0.0445
*mir-16-2*	Up	0.0445
*miR-216*	Down	0.0445
*mir-185*	Down	0.0445
*miR-18a*	Up	0.0445
*miR-17-5p*	Up	0.0448
*miR-130b*	Up	0.0448
*miR-516-3p*	Down	0.0448
*miR-106a*	Up	0.0500
*miR-21*	Up	0.0500
*miR-95*	Down	0.0551
*miR-20b*	Up	0.0833
*miR-425-5p*	Up	0.0833
*miR-181b*	Up	0.0833
*miR-181a*	Up	0.0833
*miR-135a*	Down	0.0833
*miR-181c*	Up	0.0853
*mir-203*	Down	0.0855
*miR-15a*	Up	0.0855
*miR-483*	Down	0.0855
miR-182*	Down	0.0855
*miR-20a*	Up	0.0855
*miR-181d*	Up	0.0909
*miR-98*	Up	0.0911
*mir-326*	Down	0.0986

*Up: higher expression in activated CD5^−^ B cell.

Down: higher expression in resting CD5^−^ B cell.

### Validation of miRNAs expression by quantitative RT-PCR

We validated our microrray results by quantitative RT-PCR on CD5^+^, GC and CD5^−^ activated and resting B cell mRNA samples as shown in Supplementary Figure 3. In fact, we validated 10 different miRNAs: *mir-150*, *mir-20b*, *mir-23a*, *mir-211*, *mir-15b*, *mir-21*, *mir-106a*, *mir-146a*, mir-9* and *mir-155* whose expression trends by quantitative RT-PCR highlighted the same expression trend shown by microarray analysis. The only discrepancy between RT-PCR and microarray analysis data was referred to *miR-23a* expression: this miRNA, in fact, did not show a significantly differential expression among the four B cell subsets by microarray analysis but it did show a significant upregulation by RT-PCR (*P* = 0.002) in CD5^−^ activated B cells compared to the other B cell subsets. Statistical analysis of quantitative RT-PCR results using Kruskal-Wallis test confirmed the differential expression of these miRNAs among the B cells subsets analyzed (Supplementary Figure 3).

### Validation of miR-9*, miR-29b and miR-150 on normal tonsils by *in situ* hybridization

**Figure 4:**
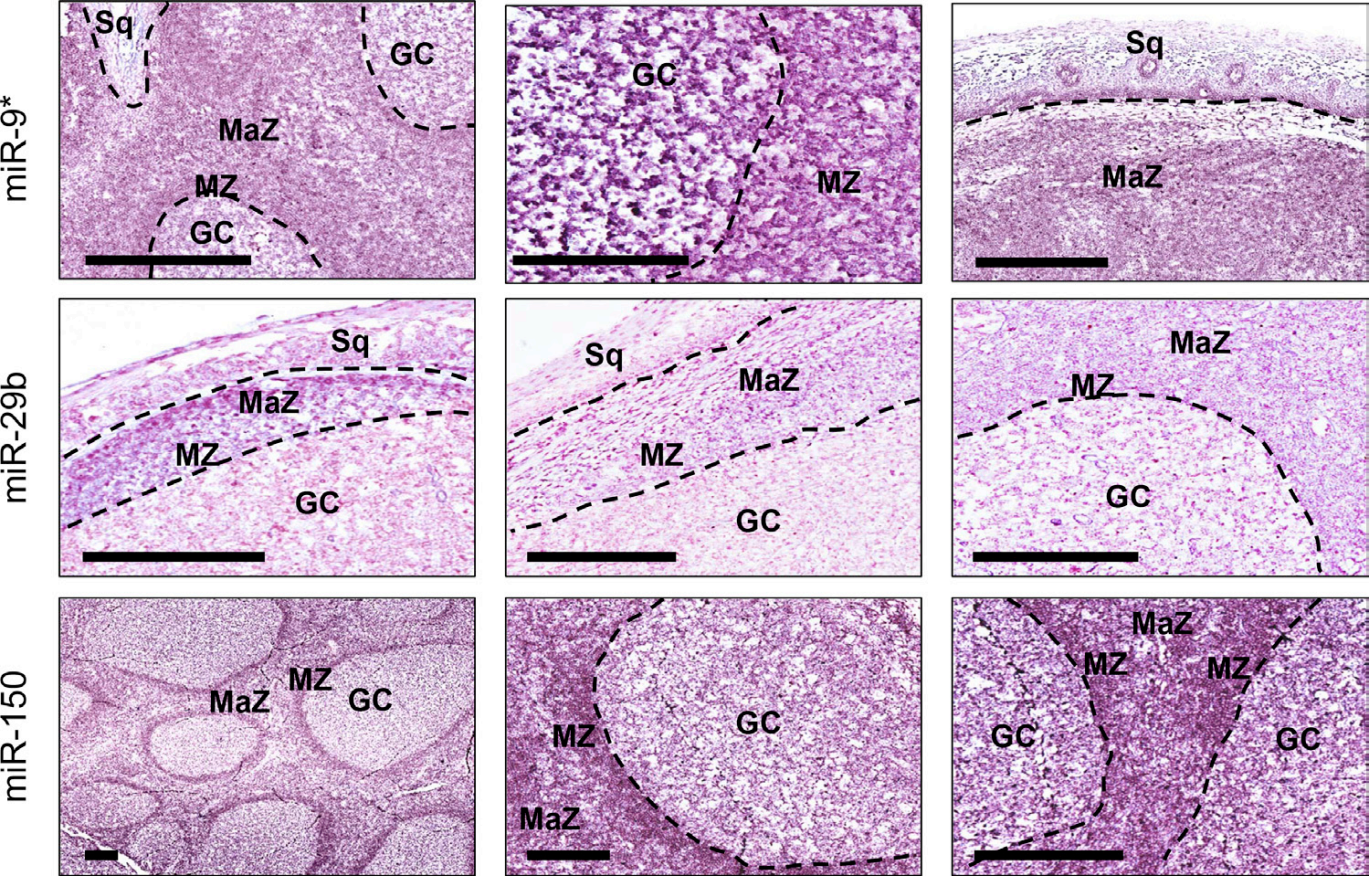
MiR-9*, miR-29b and miR-150 distribution in normal tonsillar tissue The expression of the three miRNAs was detectable in pharyngeal tonsils as a grainy blue cytoplasmic staining. MiR-9* was significantly overexpressed in germinal centers (GC), mantle zone (MZ), and subepithelial marginal zone (MaZ) in comparison to squamous epithelium (Sq). At higher magnification, it is evident a stronger expression in GC cells in comparison to MZ. MiR-29b was significantly overexpressed in both MZ and MaZ in comparison to GC, whereas miR-150 was significantly downregulated in GC in comparison to MZ and MaZ. Bar scale = 200 μm.

We validated by *in situ* hybridization miR-9*, *miR-29b* and *miR-150*, selected from microrray results. The analysis was conducted in five pharyngeal tonsils using mature miRNA labeled probes (Figure 4). The expression of the three miRNAs was detectable as a grainy blue cytoplasmic staining. MiR-9* was significantly overexpressed in germinal centers (GC), mantle zone (MZ), and subepithelial marginal zone (MaZ) in comparison to squamous epithelium (Sq). At higher magnification, it is evident a stronger expression in GC cells in comparison to MZ. *MiR-29b* was significantly overexpressed in both MZ and MaZ in comparison to GC. *MiR-150* was significantly downregulated in GC in comparison to both MZ and MaZ.

### Identification of genes regulated by differentially expressed miRNAs in the late B cell development

**Figure 5:**
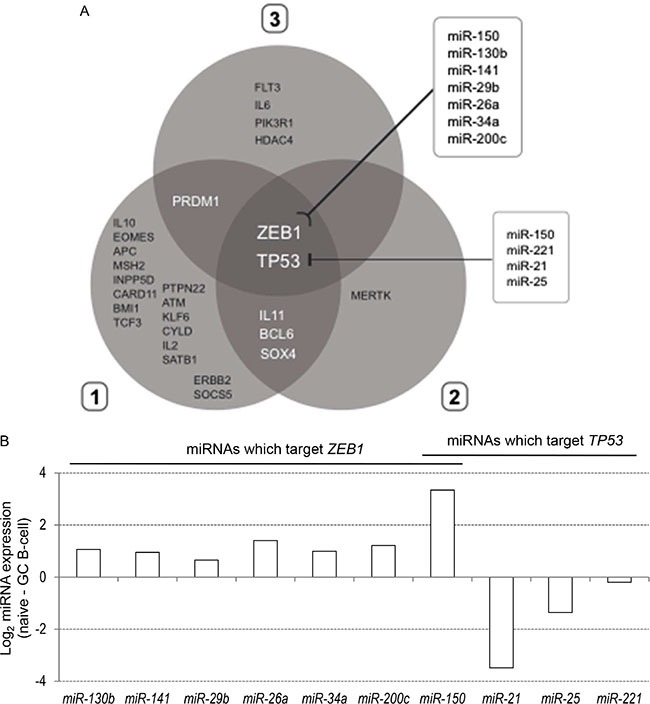
**(A)** Distribution 28 genes targeted by differentially expressed miRNAs in the three clusters reported in Figure 1. Venn diagram reports the distribution and intersection of target genes of differentially expressed miRNAs which belong to the gene ontology category “lymphocyte differentiation”. Two boxes indicate the miRNAs known to target ZEB1 and TP53. **(B)** Differential expression of miRNAs known to target ZEB1 and TP53 between naïve and GC B cells.

Using the differentially expressed miRNA profiles, we generated a list of 608 experimentally validated gene targets (Supplementary Table 3), that were filtered through the gene ontology category “lymphocyte differentiation” (GO:0030098). The distribution of 28 miRNA targets generated by the three clusters of differentially expressed miRNAs is shown in Figure 5A, where the Venn diagram highlighted two shared selective gene targets among the three miRNA clusters: *ZEB1* and *TP53* genes. In particular, we identified two selective miRNA lists: the first one, composed by *miR-150*, *miR-130b*, *miR-141*, *miR-29b, miR-26a, miR-34a* and *miR-200c*, able to target the *ZEB1* gene; and the second one, composed by *miR-150*, *miR-221*, *miR-21* and *miR-*25, able to target the *TP53* gene. The only common miRNA between the two lists is *miR-150*.

Finally, we compared the expression levels of seven *ZEB1*- and four *TP53*-targeting miRNAs in naïve and GC B cells (Figure 5B): all *ZEB1*-targeting miRNAs were expressed at higher level in naïve compared to GC B cells; while only three (*miR-221*, *miR-21* and *miR-*25) out of four *TP53-* targeting miRNAs (except *miR-150*) were upregulated in GC B cells.

### Network analysis based on the experimentally validated gene targets of differentially expressed miRNA

**Figure 6:**
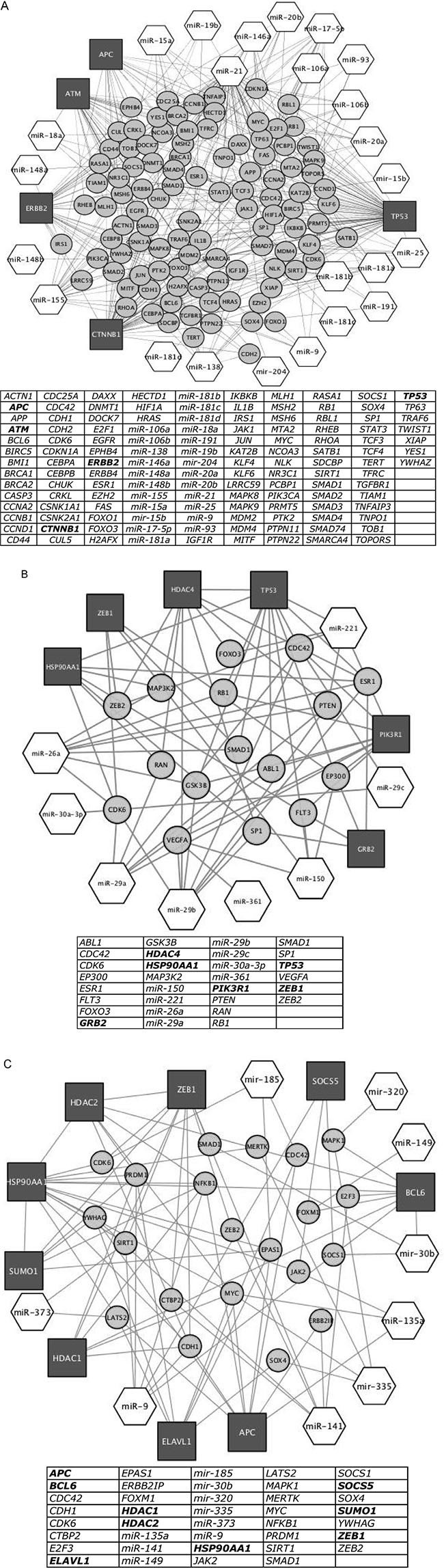
Network of proteins obtained by shortest path analysis among target genes of miRNA differentially expressed (Figure 1), their first interactor proteins and miRNAs targeting hub proteins and first interactors Dark grey square: hub proteins, that is proteins nodes of the network with higher degree; light grey circle: protein first interactors of hub proteins; white hexagon: miRNA targeting hub proteins. In tables, list of hub proteins (bold letters), first interactors of hub proteins and miRNAs targeting hub proteins and their first interactors present in the network. (A) Network of target proteins of miRNA listed in Cluster 1 of Figure 1, their first interactor proteins and miRNAs targeting hub proteins and first interactors. (B) Same representation as in 6A by using the miRNAs listed in Cluster 2 of Figure 1. (C) Same representation as in 6A by using the miRNAs listed in Cluster 3 of Figure 1.

To discover the selective cellular pathways targeted by the identified miRNAs, we performed a network analysis To infer proteins and cellular pathways modulated by miRNAs, we consulted a global intracellular protein-protein interactome (http://dp.univr.it/~laudanna/LCTST/downloads/index.html). For each cluster of differentially expressed miRNAs, we identified the nodes involved in the shortest paths among experimentally validated miRNA targets listed in Figure 5A. We considered the proteins with highest degree (hubs) as the most perturbed targets by the biological processes under investigation. Finally we generated for each miRNA cluster a sub-network including only the hubs and the paths that are connected to the differentially expressed miRNAs. Using this representation, we highlighted the relationship between miRNA-related changes and potential protein targets. We identified a cluster 1-related sub-network that was composed by 133 items, including the 5 main hubs (*APC*, *ATM*, *CTNNB1*, *ERBB2*, *TP53*); a cluster 2-related sub-network that contained 30 items, including the 6 hubs (*GRB2*, *HDAC4*, *HSP90AA1*, *PIK3R1*, *TP53* and *ZEB1*) and, finally, a cluster 3-related sub-network that included 9 hubs (*APC*, *BCL6*, *ELAVL1*, *HDAC1*, *HDAC2*, *HSP90AA1*, *SOCS5*, *SUMO1* and *ZEB1*) (Figure 6A–6C respectively).

### Gene ontology analysis

To better define the miRNA-related regulatory potential functions on controlling B cell biological modifications during the GC reaction, we analyzed the gene annotation of all primary protein-interactors connected to the shortest paths analyses. We identified 52 Panther pathways and 49 Panther hallmarks that were significantly over-represented as reported in Supplementary Table 4 and Supplementary Table 5, respectively. The top 10 enriched cellular pathways were: “CCKR signaling map“ (*P* = 1.66 × 10^−64^); “Gonadotropin releasing hormone receptor pathway“ (*P* = 1.44 × 10^−41^); “Angiogenesis“ (*P* = 7.15 × 10^−39^); “EGF receptor signaling pathway“ (*P* = 7.92 × 10^−36^); “Apoptosis signaling pathway“ (*P* = 5.86 × 10^−35^); “Interleukin signaling pathway“ (*P* = 6.91 × 10^−35^); “B cell activation” (*P* = 1.73 × 10^−32^); “T cell activation“ (*P* = 1.14 × 10^−30^); “Inflammation mediated by chemokine and cytokine signaling pathway” (*P* = 8.44 × 10^−28^); “Integrin signalling pathway“ (*P* = 1.04 × 10^−27^) (Supplementary Table 4).

The top 10 enriched hallmarks categories were: “Genes up-regulated during transplant rejection” (*P* = 7.09 × 10^−57^); “Genes up-regulated by activation of the PI3K/AKT/mTOR pathway” (*P* = 7.47 × 10^−46^); “Genes involved in the G2/M checkpoint, as in progression through the cell division cycle“ (*P* = 7.91 × 10^−46^); “Genes important for mitotic spindle assembly“ (*P* = 1.66 × 10^−43^); “A subcluster of genes regulated by MYC - version 1 (v1)” (*P* = 12.14 × 10^−42^); “Genes encoding cell cycle related targets of *E2F* transcription factors“ (*P* = 1.85 × 10^−35^); “Genes mediating programmed cell death (apoptosis) by activation of caspases“ (*P* = 3.82 × 10^−31^); “Genes regulated by NF-kB in response to TNF [GeneID = 7124]”; (*P* = 3.87 × 10^−31^); “Genes involved in p53 pathways and networks“ (*P* = 4.87 × 10^−29^); “Genes up-regulated in response to IFNG [GeneID = 3458]” (*P* = 5,00 × 10^−28^) (Supplementary Table 5).

## DISCUSSION

MiRNAs establish regulatory networks by intersecting and affecting multiple signaling pathways [22, 23]. Trying to disclose the miRNA-related regulatory actions during the late phase of B cell differentiation, we firstly identified miRNAs profiles of B cell subsets in different maturative phases and their prospective relationships in association to the cell machinery. Using this strategy, we identified molecular pathways that are modulated by selective miRNAs.

Microarray or quantitative RT-PCR analyses have already assessed the miRNAs involved in the late differentiation of B cells [12–14]. The results reported in literature, however, are partially conflicting about both the number of differentially expressed miRNAs and their expression levels. In this complex scenario our data achieved some essential goals that can be summarized in the following points: 1) the cell transition from immature to mature B lymphocytes is strictly characterized by the modulation of 107 miRNAs: 33 miRNAs change their expression during B cell passage from blood to peripheral lymphoid organs; 2) the three main B cell populations in tonsils (naïve, GC and SE B cells) involved in the GC reaction showed distinct expression profiles: 48 miRNAs are differentially expressed during the GC reaction and they are segregated in three clusters according to the sequential maturative stages; 3) among the 48 differentially expressed miRNAs, we identified 8 new original miRNAs (*mir-323*, *mir-138*, mir-9*, *mir-211*, *mir-149*, *mir-373*, *mir-135a* and *mir-184*) that were not previously reported as discriminative miRNAs during B cell development; 4) the expression of 34 miRNAs are able to discriminate between resting and activated CD5^−^ SE B cells; moreover, miRNAs from resting CD5^−^ B cell better resemble miRNAs from naïve B cell rather than miRNAs from activated CD5^−^ B cell ; 5) during the late phase of B cell differentiation *ZEB1* and *TP53* are the most influenced targets by the differentially expressed miRNAs; 6) *APC*, *ATM*, *CTNNB1*, *ERBB2*, *TP53*, *BCL6*, *SOCS5*, *HDAC4*, *HDAC2*, *HDAC1*, *GRB2*, *PIK3R1*, *HSP90AA1*, *ZEB1*, *SUMO1* and *ELAVL1* are the main hub proteins that are controlled by the identified miRNAs; 7) gene ontology analysis showed that miRNAs controls core signaling pathways and essential for B cell functions.

Moreover, our new identified differentially expressed miRNAs are related to other specific aspects of B cell biology. We discovered that *miR-323* expression was reduced in activated mature B cells compared to naïve and GC B cells. This miRNA was reported as a biomarker of immune and inflammatory response, since it is an activator of the Wnt/Cadherin signaling pathway and it induces cytokine expression and tissue metalloproteases [24] in rheumathoid arthritis. Moreover, *miR-323* was recently described to target *EED*, a component of the polycomb repressive complex 2 (PRC2) [25], that plays an essential role in GC formation; in fact, the modulation of *miR-323* in the follicles was reported as a critical and relevant event of the adaptive immune response [26]. Recently, *mir-138* was shown to target acyl protein thioesterases (*LYPLA1* and *LYPLA2*) able to regulate the palmitoylation of CD95 and apoptosis in chronic lymphoblastic leukemia [27]. *Mir-138* was found upregulated in follicular lymphoma but downregulated in the GC subtype of diffuse large B cell lymphoma [28] [29]. The over-expression of *mir-211* has been already associated with lymphoma progression [30]. By contrast, specific alterations of Mir-9* expression were never reported in normal B cell populations, even if this miRNA is a selective marker of follicular lymphomas [28]. Similarly, the differential expression levels of *mir-149* were never described during physiological B cell development even if its downregulation have been associated to both diffuse large and follicular lymphoma [28, 31]. Finally, the differential expression of *mir-373* was reported in several neoplastic diseases except in lymphomas [32]; on the contrary, both the *mir-135a* downregulation (able to target *JAK2*) and *miR-135a* overexpression were associated to relapse of Hodgkin's [33] and follicular and diffuse large B cell lymphomas [29, 31]. Finally, the downregulation of *mir-184* was reported in marginal zone lymphoma [34].

A very interesting observation is related to *miR-185* expression pattern: published results, in fact, demonstrated that this miRNA was upregulated in mouse GC B cells and downregulated in human GC B cells after stimulation of naïve B cells with lipopolysaccharide antigen [21, 35]. *MiR-185* is able to switch off the *BTK* kinase, that plays a critical role in controlling the positive and negative selection of autoreactive B cells in mice [36], by influencing the B cell receptor signaling pathway [21]. Our data confirmed that *miR-185* is downregulated in human GC B cells. In fact, based on these evidences, we speculate that *miR-185* level, in connection with other regulatory mechanisms having different weights in human and mice, participates to the fine tuning of the threshold for the selection of autoreactive B cells.

The SE niche of tonsils is populated by mature CD5^−^ B cells comprising small resting B lymphocytes and activated plasmablasts with peculiar phenotypic characteristics [37, 38]. According to the classical view of the primary immune response, the non-cycling hypermutated class-switched mature B cells are the progeny of adjacent positively antigen-selected GC B cells [17, 39]. Instead, the nature of SE resting cells not-class switched, IgV gene unmutated and insensitive to T helper-mediated activation, remains elusive [17, 40, 41]. In mice, the memory pool contains stable fractions of unmutated and mutated IgV gene cells [19]. In humans, the memory B cell compartment is not totally well defined and the existence of a T helper- and GC-independent pathway of maturation of B cells is still controversial [41]. According to our data, activated and resting mature B cells have unique expression profiles of miRNAs. These data reinforced the hypothesis that these two B cell populations are truly two distinct entities: in fact, the miRNA profile of resting CD5^−^ B cells better resembled that of naive CD5^+^ B cells rather than that of activated CD5^−^ B cells. In addition, the SE resting cells showed the upregulation of a set of miRNAs tipically upregulated in activated mature B cells compared to GC B cells. Therefore, our results clearly demonstrated that resting CD5^−^ B cells could be defined as a specific subpopulation of CD5^+^ B cells that have exceed the GC selection and partially acquired the features of mature B cells.

In the GC reaction, the balance between positive and negative inputs is critical for an effective physiological response to antigens [42]. We identified the *ZEB1* and *TP53* genes as main targets of differentially expressed miRNAs during the late B cell differentiation. *ZEB1*–targeting miRNAs are downregulated on GC B cells, suggesting a possible biological effect on B cells promoted by the increased *ZEB1* expression level. *ZEB1* is involved both in cell reprogramming and in the epithelial-to-mesenchymal transition; but its role in B cell development is still unclear. In mice, Zeb1 expression is required for GC formation and memory B cell response. The enforced Zeb1 expression after the B cell receptor (BCR)-antigen binding promoted both proliferation and altered apoptotic death of naïve B cells [43]. Moreover, Zeb1 plays a critical role in regulating the functions of *BCL6* that is a rheostat factor in controlling B cell fate. In fact, *ZEB1* binds the E-box motif that controls the *BCL6* and *SMAD* genes regulating the GC reaction and the TGF-β1 signaling pathway, respectively [44]. In humans, Zeb1 is almost absent in quiescent B cells of the mantle zone but it is expressed in proliferating GC B cells of tonsils (http://www.proteinatlas.org/ENSG00000148516-ZEB1/tissue/tonsil). High Zeb1 expression was also associated with adverse overall survival in diffuse large and mantle B cell lymphomas [45–47]. The higher Zeb1 expression levels in malignant lymphomas suggest its involvement during B cell differentiation either at early stages or after BCR activation. Therefore, all these evidences indicate that miRNAs could contribute to set Zeb1 levels and action timing during early cellular events following BCR activation.

*TP53* exerts a central role in maintaining cell homeostasis following genotoxic insults. *BCL6* modulates the B cell response inducing tolerance to DNA damage-induced apoptosis by suppressing *TP53* in GC B cells, [48]; while *P53* controls the cell cycle at two distinctive checkpoints (G1/S and G2/M) by the regulation of *miR-107*, *miR-145*, *miR-34*, and of the miRNA clusters *miR-15a/miR-16* and *miR-192/miR-194/miR-215*, able to target many cell cycle-related genes [49]. An increased level of *TP53*-targeting miRNAs in GC B cells such as *miR-21*, *miR-25* and *miR-200c* could promote an additional control level of *TP53* expression after the activation of the DNA damage response.

By expanding our analysis through the protein interactome, we identified hub proteins under the control of modulated miRNAs during the late B cell differentiation. These target proteins are involved in different pathways suggesting that these miRNAs can regulate several signaling networks that are activated during B cell development. We identified some hub proteins that have been already reported as key player in main signaling pathways, but also some specific hubs that were recently directly implicated in the B cell development. In particular, we identified *ELAVL1* (also known as HuR), that plays an essential role in the GC reaction and class-switched antibodies production [50]. We demonstrated that *miR-9*, which targets *ELAVL1*, decreased its expression in the transition from naïve to GC and mature B cells, suggesting that miRNA could control HuR expression levels.

In conclusion, we described the modulation of miRNAs and the involvement of underlying miRNA targets and cellular functions in the late B cell maturation. Our findings complement the results reported in previous studies and offer new hints for further functional studies addressing the comprehension of the role played by miRNAs in B lymphocyte biology.

## MATERIALS AND METHODS

### Samples

Twenty-nine CD19^+^ normal B cell samples were studied. Four CD19^+^ B cell samples were obtained from the peripheral blood of four healty donors and twenty-five CD19^+^ normal B cell samples were prepared from nonpathological tonsils after surgical removal at the National Institute for Cancer Research of Genova. The materials used have been collected under Program 1-IC01 protocol IC/01/LLC/001 13/12/2001, renewed 28/11/2013. The protocols, which concerned the collection of the informed consent of patients, were approved by the local ethics committee of the IRCCS Azienda Ospedaliera Integrata San Martino – IST Istituto Nazionale per la Ricerca sul Cancro (Genova). Among B cell samples from tonsils, two were naïve CD5^+^ B cells (each sample was a pool of five different donors), twelve samples were CD23^−^CD39^−^ GC B cells; eleven samples were CD5^−^ subepithelial mature B cells. Among the eleven SE CD5^−^ B cell samples, four samples were SE CD5^−^ IgV hypermutated activated B cells and seven samples were SE CD5^−^ not IgV hypermutated resting B cells. Subepithelial CD5^−^ B cells of tonsils are equivalent to the marginal zone B cells in other peripheral lymphoid organs [17].

### Cell separations and RNA extraction

Pure populations of B lymphocytes were isolated by antibody-conjugated magnetic beads sorting according to established protocols. CD19^+^ cells were isolated from the peripheral blood of healthy donors by positive selection with anti-CD19 antibody magnetic beads-conjugated and magnetic sorting. Four CD19^+^ B cell populations, naïve B cells (CD5^+^), GC B cells (CD23^−^CD39^−^) and two populations of CD5^−^ B cells, SE activated IgD^+^ (IgV gene mutated) and SE resting IgD^−^ (IgV gene unmutated), were isolated from tonsils as previously described [17]. Purity of B lymphocytes was at least 95%, according to cytofluorometer analysis. Total RNA was isolated from cells using TRIZOL reagent (Invitrogen) according to manufacturer protocol. RNA concentration and integrity was determined respectively by spectrophotometer and agarose gel separation

### Microarrays

MicroRNA labeling and hybridization were performed using 5 mgmicrograms total RNA, as described [51]. We used a multi-species microarray platform containing 2284 probes, 1256 for human and 1028 for mouse targets, respectively. A total of 353 human mature or pre-miRNA were detectable by the microarray. Each human target was matched by at least two probes, with an average of 4.3 probes for each target. Hybridization signals were detected with Streptavidin-Alexa647 conjugate and scanned images (Axon 4000B) were quantified using the Genepix 6.0 software (Axon Instruments).

### Data analysis

Expression data from microarrays were normalized and transformed using the *vsn* package for R. The spots were subsequently classified based on their target sequence regardless of the original designation. The expression measures for probes matching the same miR sequence were summarized using the *medpolish* algorithm, in order to obtain a unique expression figure for each target. Clustering analysis was performed using the *hclust* function and the inverse Pearson correlation as a distance metric, for both genes and arrays. All the clusters were visualized using the Java TreeView software (http://jtreeview.sourceforge.net). To select differentially expressed genes, we performed either anova or *t*-tests. To take into account multiple hypothesis testing, the FDR (false discovery rate) was calculated using the *qvalue* package for R. All the genes with a FDR less than 0.01 were used in subsequent analyses. All the calculations were performed using the R statistical software (http://www.r-project.org).

### Evaluation of miRNAs expression by quantitative RT-PCR

For each sample and for each miRNA, 5 ng of total RNA was converted to cDNA by TaqMan MicroRNA Reverse Transcription kit (Applied Biosystems) with the miRNA specific primer contained in the TaqMan MicroRNA assays (Applied Biosystems). MiRNA expression was evaluated in 10 μL total volume by quantitative RT-PCR following the manufacturer protocol in the presence of 1× TaqMan Universal Master Mix (Applied Biosystems) on a 7900HT SDS instrument (Applied Biosystems). Expression differences of miRNAs among samples were determined by the comparative method according to User Bulletin #2 (Applied Biosystems) using the noncoding RNA RNU44 and RNU6B as reference gene.

### MiRNA *in situ* hybridization analysis

Locked nucleic acid (LNA) probes with complementarity to miR-9*, *miR-29b*, and *miR-150* were labelled with 5′-biotin and synthesised using Exiqon (Vedbaek, Denmark). Tissue sections were digested with ISH protease 1 (Ventana Medical Systems, Milan, Italy) and ISH was performed as we previously described (PMID:27618837). Positive (U6; Exiqon) and negative scrambled LNA probes (Exiqon) were used as controls. Only cytoplasmic miRNA staining was retained for scoring purposes.

### Network analysis

Experimentally validated target genes (only target genes marked as “strong evidence”) of differentially expressed miRNAs were retrieved from MirTarBase 4.5 database [52]. Among the predicted targets, only those included in the GO category “lymphocyte differentiation” (GO:0030098) were kept. Using the filtered targets and a database of protein interactions (http://dp.univr.it/~laudanna/LCTST/downloads/index.html), comprising 14642 proteins and 270062 edges, we generated subnetworks which included the shortest paths joining the primary targets. Shortest paths were calculated with PeSca application 3.0 version implemented in Cytoscape 3.0 [53].

### Gene ontology and pathway analysis

A comprehensive list of first interactors of genes which are nodes of the shortest paths was submitted to Panther (Protein ANalysis THrough Evolutionary Relationships) Classification System at http://pantherdb.org/) for evaluation of enrichment of annotations for functional classification and cellular pathways [54, 55]. For gene ontology and pathway analysis, a significant value threshold of 0.05 was applied.

## SUPPLEMENTARY MATERIALS FIGURES AND TABLES





## References

[1] Cooper MD (2015). The early history of B cells. Nat Rev Immunol.

[2] Nescakova Z, Bystricky S (2011). B cells - ontogenesis and immune memory development. Gen Physiol Biophys.

[3] Matthias P, Rolink AG (2005). Transcriptional networks in developing and mature B cells. Nat Rev Immunol.

[4] Shen Y, Iqbal J, Xiao L, Lynch RC, Rosenwald A, Staudt LM, Sherman S, Dybkaer K, Zhou G, Eudy JD, Delabie J, McKeithan TW, Chan WC (2004). Distinct gene expression profiles in different B-cell compartments in human peripheral lymphoid organs. BMC Immunol.

[5] Fernando TR, Rodriguez-Malave NI, Rao DS MicroRNAs in B cell development and malignancy. J Hematol Oncol.

[6] De Tullio G, De Fazio V, Sgherza N, Minoia C, Serrati S, Merchionne F, Loseto G, Iacobazzi A, Rana A, Petrillo P, Silvestris N, Iacopino P, Guarini A (2014). Challenges and opportunities of microRNAs in lymphomas. Molecules.

[7] de Yebenes VG, Bartolome-Izquierdo N, Ramiro AR (2013). Regulation of B-cell development and function by microRNAs. Immunol Rev.

[8] Koralov SB, Muljo SA, Galler GR, Krek A, Chakraborty T, Kanellopoulou C, Jensen K, Cobb BS, Merkenschlager M, Rajewsky N, Rajewsky K (2008). Dicer ablation affects antibody diversity and cell survival in the B lymphocyte lineage. Cell.

[9] Rao DS, O’Connell RM, Chaudhuri AA, Garcia-Flores Y, Geiger TL, Baltimore D (2010). MicroRNA-34a perturbs B lymphocyte development by repressing the forkhead box transcription factor Foxp1. Immunity.

[10] Xiao C, Calado DP, Galler G, Thai TH, Patterson HC, Wang J, Rajewsky N, Bender TP, Rajewsky K (2007). MiR-150 controls B cell differentiation by targeting the transcription factor c-Myb. Cell.

[11] Danger R, Braza F, Giral M, Soulillou JP, MicroRNAs Brouard S (2014). Major Players in B Cells Homeostasis and Function. Front Immunol.

[12] Malumbres R, Sarosiek KA, Cubedo E, Ruiz JW, Jiang X, Gascoyne RD, Tibshirani R, Lossos IS (2009). Differentiation stage-specific expression of microRNAs in B lymphocytes and diffuse large B-cell lymphomas. Blood.

[13] Tan LP, Wang M, Robertus JL, Schakel RN, Gibcus JH, Diepstra A, Harms G, Peh SC, Reijmers RM, Pals ST, Kroesen BJ, Kluin PM, Poppema S (2009). miRNA profiling of B-cell subsets: specific miRNA profile for germinal center B cells with variation between centroblasts and centrocytes. Lab Invest.

[14] Zhang J, Jima DD, Jacobs C, Fischer R, Gottwein E, Huang G, Lugar PL, Lagoo AS, Rizzieri DA, Friedman DR, Weinberg JB, Lipsky PE, Dave SS (2009). Patterns of microRNA expression characterize stages of human B-cell differentiation. Blood.

[15] Garraud O, Borhis G, Badr G, Degrelle S, Pozzetto B, Cognasse F, Richard Y (2012). Revisiting the B-cell compartment in mouse and humans: more than one B-cell subset exists in the marginal zone and beyond. BMC Immunol.

[16] Song H, Cerny J (2003). Functional heterogeneity of marginal zone B cells revealed by their ability to generate both early antibody-forming cells and germinal centers with hypermutation and memory in response to a T-dependent antigen. J Exp Med.

[17] Dono M, Zupo S, Leanza N, Melioli G, Fogli M, Melagrana A, Chiorazzi N, Ferrarini M (2000). Heterogeneity of tonsillar subepithelial B lymphocytes, the splenic marginal zone equivalents. J Immunol.

[18] Dono M, Burgio VL, Colombo M, Sciacchitano S, Reverberi D, Tarantino V, Cutrona G, Chiorazzi N, Ferrarini M (2007). CD5+ B cells with the features of subepithelial B cells found in human tonsils. Eur J Immunol.

[19] Kaji T, Ishige A, Hikida M, Taka J, Hijikata A, Kubo M, Nagashima T, Takahashi Y, Kurosaki T, Okada M, Ohara O, Rajewsky K, Takemori T (2012). Distinct cellular pathways select germline-encoded and somatically mutated antibodies into immunological memory. J Exp Med.

[20] Caraux A, Klein B, Paiva B, Bret C, Schmitz A, Fuhler GM, Bos NA, Johnsen HE, Orfao A, Perez-Andres M (2010). Circulating human B and plasma cells. Age-associated changes in counts and detailed characterization of circulating normal CD138− and CD138+ plasma cells. Haematologica.

[21] Belver L, de Yebenes VG, Ramiro AR (2010). MicroRNAs prevent the generation of autoreactive antibodies. Immunity.

[22] Lui PY, Jin DY, Stevenson NJ (2015). MicroRNA: master controllers of intracellular signaling pathways. Cell Mol Life Sci.

[23] Suzuki HI, Miyazono K (2011). Emerging complexity of microRNA generation cascades. J Biochem.

[24] Xu T, Li L, Huang C, Li X, Peng Y, Li J (2014). MicroRNA-323-3p with clinical potential in rheumatoid arthritis, Alzheimer's disease and ectopic pregnancy. Expert Opin Ther Targets.

[25] Zhang Y, Teng F, Luo GZ, Wang M, Tong M, Zhao X, Wang L, Wang XJ, Zhou Q (2013). MicroRNA-323-3p regulates the activity of polycomb repressive complex 2 (PRC2) via targeting the mRNA of embryonic ectoderm development (Eed) gene in mouse embryonic stem cells. J Biol Chem.

[26] Lee SC, Miller S, Hyland C, Kauppi M, Lebois M, Di Rago L, Metcalf D, Kinkel SA, Josefsson EC, Blewitt ME, Majewski IJ, Alexander WS (2015). Polycomb repressive complex 2 component Suz12 is required for hematopoietic stem cell function and lymphopoiesis. Blood.

[27] Berg V, Rusch M, Vartak N, Jungst C, Schauss A, Waldmann H, Hedberg C, Pallasch CP, Bastiaens PI, Hallek M, Wendtner CM, Frenzel LP (2015). miRs-138 and -424 control palmitoylation-dependent CD95-mediated cell death by targeting acyl protein thioesterases 1 and 2 in CLL. Blood.

[28] Di Lisio L, Sanchez-Beato M, Gomez-Lopez G, Rodriguez ME, Montes-Moreno S, Mollejo M, Menarguez J, Martinez MA, Alves FJ, Pisano DG, Piris MA, Martinez N (2012). MicroRNA signatures in B-cell lymphomas. Blood Cancer J.

[29] Lawrie CH, Chi J, Taylor S, Tramonti D, Ballabio E, Palazzo S, Saunders NJ, Pezzella F, Boultwood J, Wainscoat JS, Hatton CS (2009). Expression of microRNAs in diffuse large B cell lymphoma is associated with immunophenotype, survival and transformation from follicular lymphoma. J Cell Mol Med.

[30] Chitnis NS, Pytel D, Bobrovnikova-Marjon E, Pant D, Zheng H, Maas NL, Frederick B, Kushner JA, Chodosh LA, Koumenis C, Fuchs SY, Diehl JA (2012). miR-211 is a prosurvival microRNA that regulates chop expression in a PERK-dependent manner. Mol Cell.

[31] Roehle A, Hoefig KP, Repsilber D, Thorns C, Ziepert M, Wesche KO, Thiere M, Loeffler M, Klapper W, Pfreundschuh M, Matolcsy A, Bernd HW, Reiniger L, Merz H, Feller AC (2008). MicroRNA signatures characterize diffuse large B-cell lymphomas and follicular lymphomas. Br J Haematol.

[32] Wei F, Cao C, Xu X, Wang J (2015). Diverse functions of miR-373 in cancer. J Transl Med.

[33] Navarro A, Diaz T, Martinez A, Gaya A, Pons A, Gel B, Codony C, Ferrer G, Martinez C, Montserrat E, Monzo M (2009). Regulation of JAK2 by miR-135a: prognostic impact in classic Hodgkin lymphoma. Blood.

[34] Iqbal J, Shen Y, Liu Y, Fu K, Jaffe ES, Liu C, Liu Z, Lachel CM, Deffenbacher K, Greiner TC, Vose JM, Bhagavathi S, Staudt LM (2012). Genome-wide miRNA profiling of mantle cell lymphoma reveals a distinct subgroup with poor prognosis. Blood.

[35] Yu M, Liang W, Wen S, Zhao T, Zhu MX, Li HH, Long Q, Wang M, Cheng X, Liao YH, Yuan J (2014). EphB2 contributes to human naive B-cell activation and is regulated by miR-185. FASEB J.

[36] Kil LP, de Bruijn MJ, van Nimwegen M, Corneth OB, van Hamburg JP, Dingjan GM, Thaiss F, Rimmelzwaan GF, Elewaut D, Delsing D, van Loo PF, Hendriks RW (2012). Btk levels set the threshold for B-cell activation and negative selection of autoreactive B cells in mice. Blood.

[37] Hsu SM (1985). Phenotypic expression of B lymphocytes. III. Marginal zone B cells in the spleen are characterized by the expression of Tac and alkaline phosphatase. J Immunol.

[38] Dono M, Burgio VL, Tacchetti C, Favre A, Augliera A, Zupo S, Taborelli G, Chiorazzi N, Grossi CE, Ferrarini M (1996). Subepithelial B cells in the human palatine tonsil. I. Morphologic, cytochemical and phenotypic characterization. Eur J Immunol.

[39] Tierens A, Delabie J, Michiels L, Vandenberghe P, De Wolf-Peeters C (1999). Marginal-zone B cells in the human lymph node and spleen show somatic hypermutations and display clonal expansion. Blood.

[40] Inamine A, Takahashi Y, Baba N, Miyake K, Tokuhisa T, Takemori T, Abe R (2005). Two waves of memory B-cell generation in the primary immune response. Int Immunol.

[41] Seifert M, Przekopowitz M, Taudien S, Lollies A, Ronge V, Drees B, Lindemann M, Hillen U, Engler H, Singer BB, Kuppers R Functional capacities of human IgM memory B cells in early inflammatory responses and secondary germinal center reactions.

[42] De Silva NS, Klein U (2015). Dynamics of B cells in germinal centres. Nat Rev Immunol.

[43] Arnold CN, Pirie E, Dosenovic P, McInerney GM, Xia Y, Wang N, Li X, Siggs OM, GB Karlsson Hedestam, Beutler B A forward genetic screen reveals roles for Nfkbid, Zeb1, and Ruvbl2 in humoral immunity.

[44] Papadopoulou V, Postigo A, Sanchez-Tillo E, Porter AC, Wagner SD (2010). ZEB1 and CtBP form a repressive complex at a distal promoter element of the BCL6 locus. Biochem J.

[45] Huang WT, Kuo SH, Cheng AL, Lin CW (2014). Inhibition of ZEB1 by miR-200 characterizes Helicobacter pylori-positive gastric diffuse large B-cell lymphoma with a less aggressive behavior. Mod Pathol.

[46] Lemma S, Karihtala P, Haapasaari KM, Jantunen E, Soini Y, Bloigu R, Pasanen AK, Turpeenniemi-Hujanen T, Kuittinen O (2013). Biological roles and prognostic values of the epithelial-mesenchymal transition-mediating transcription factors Twist, ZEB1 and Slug in diffuse large B-cell lymphoma. Histopathology.

[47] Sanchez-Tillo E, Fanlo L, Siles L, Montes-Moreno S, Moros A, Chiva-Blanch G, Estruch R, Martinez A, Colomer D, Gyorffy B, Roue G, Postigo A (2014). The EMT activator ZEB1 promotes tumor growth and determines differential response to chemotherapy in mantle cell lymphoma. Cell Death Differ.

[48] Phan RT, Dalla-Favera R (2004). The BCL6 proto-oncogene suppresses p53 expression in germinal-centre B cells. Nature.

[49] Hermeking H (2012). MicroRNAs in the p53 network: micromanagement of tumour suppression. Nat Rev Cancer.

[50] Diaz-Munoz MD, Bell SE, Fairfax K, Monzon-Casanova E, Cunningham AF, Gonzalez-Porta M, Andrews SR, Bunik VI, Zarnack K, Curk T, Heggermont WA, Heymans S, Gibson GE (2015). The RNA-binding protein HuR is essential for the B cell antibody response. Nat Immunol.

[51] Liu CG, Calin GA, Meloon B, Gamliel N, Sevignani C, Ferracin M, Dumitru CD, Shimizu M, Zupo S, Dono M, Alder H, Bullrich F, Negrini M An oligonucleotide microchip for genome-wide microRNA profiling in human and mouse tissues.

[52] Hsu SD, Tseng YT, Shrestha S, Lin YL, Khaleel A, Chou CH, Chu CF, Huang HY, Lin CM, Ho SY, Jian TY, Lin FM, Chang TH (2014). miRTarBase update 2014: an information resource for experimentally validated miRNA-target interactions. Nucleic Acids Res.

[53] Shannon P, Markiel A, Ozier O, Baliga NS, Wang JT, Ramage D, Amin N, Schwikowski B, Ideker T (2003). Cytoscape: a software environment for integrated models of biomolecular interaction networks. Genome Res.

[54] Thomas PD, Campbell MJ, Kejariwal A, Mi H, Karlak B, Daverman R, Diemer K, Muruganujan A, Narechania A (2003). PANTHER: a library of protein families and subfamilies indexed by function. Genome Res.

[55] Mi H, Dong Q, Muruganujan A, Gaudet P, Lewis S, Thomas PD (2010). PANTHER version 7: improved phylogenetic trees, orthologs and collaboration with the Gene Ontology Consortium. Nucleic Acids Res.

