# Supplementary material for: Identification of microRNAs implicated in the late differentiation stages of normal B cells suggests a central role for miRNA targets ZEB1 and TP53
Source: Oncotarget. 2017 Jan 17;8(7):11809–26. doi: 10.18632/oncotarget.14683 (PMC5355306; doi:10.18632/oncotarget.14683)
Supplement: Supplementary file 1 [file oncotarget-08-11809-s001.pdf]

Identification of microRNAs implicated in the late differentiation stages of normal B cells suggests a central role for miRNA targets *ZEB1* and *TP53*

Supplementary Materials

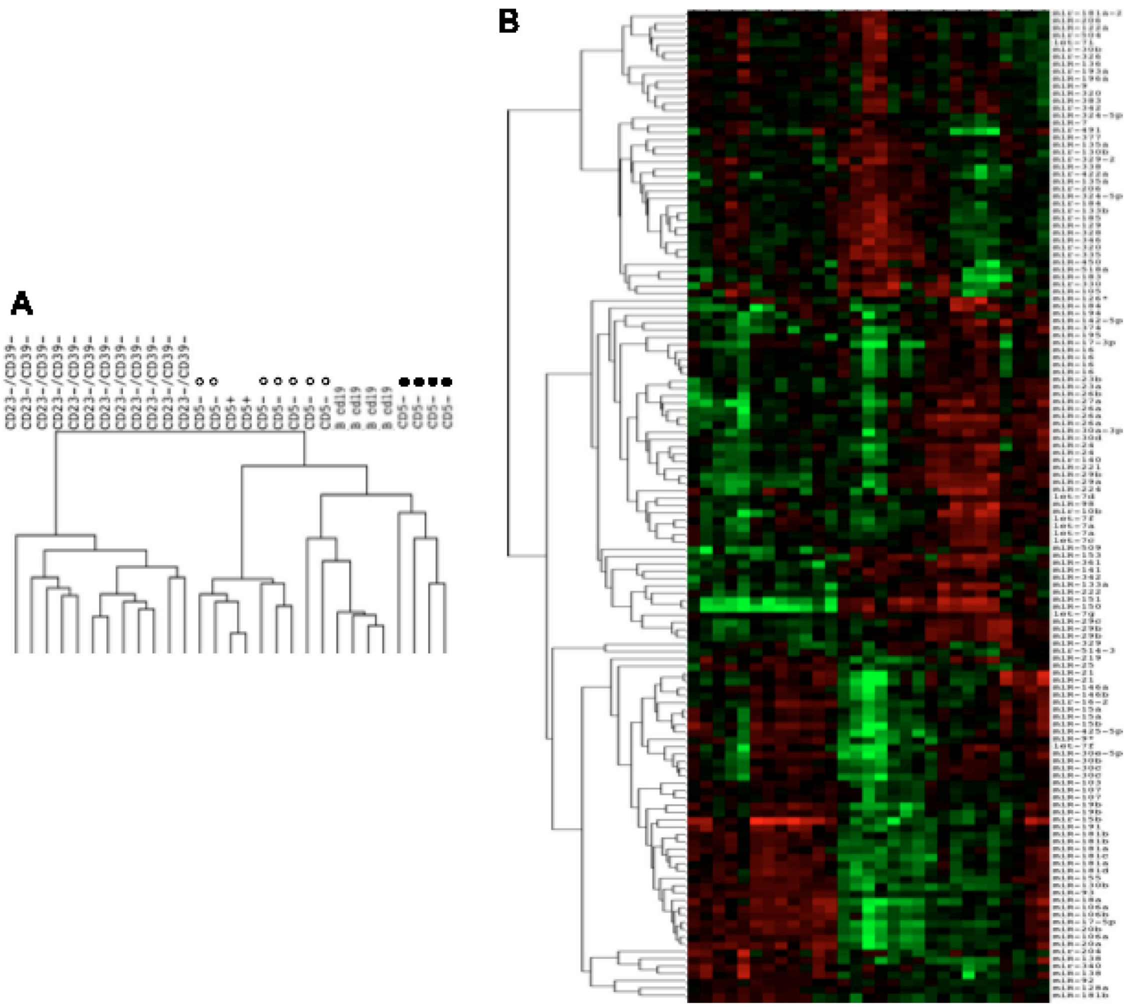

Supplementary Figure 1: Expression profile of miRNAs in $CD19^+$ cell subsets representing different stages of B cell maturation in blood and tonsils. $CD19^+$: $CD19^+$ B cells from blood; $CD5^+$: naïve B cells from tonsils; $CD23^-/CD39^-$: germinal centre (GC) B cells from tonsils; $CD5^-$: subepithelial (SE) mature B cells from tonsils, subdivided in $CD5^-$ resting and $CD5^-$ activated B cells. (**A**) Array tree of 25 samples representing different stages of maturation of the B cells based on the expression levels of miRNAs. White circles: $CD5^-$ SE resting B cells; black circles: $CD5^-$ SE activated B cells. (**B**) The heat map describes the expression levels of 107 differentially expressed miRNAs in 29 samples owning to five B cell subsets (FDR 1%). Red: higher expression (log2, +4); green: lower expression (log2, −4).

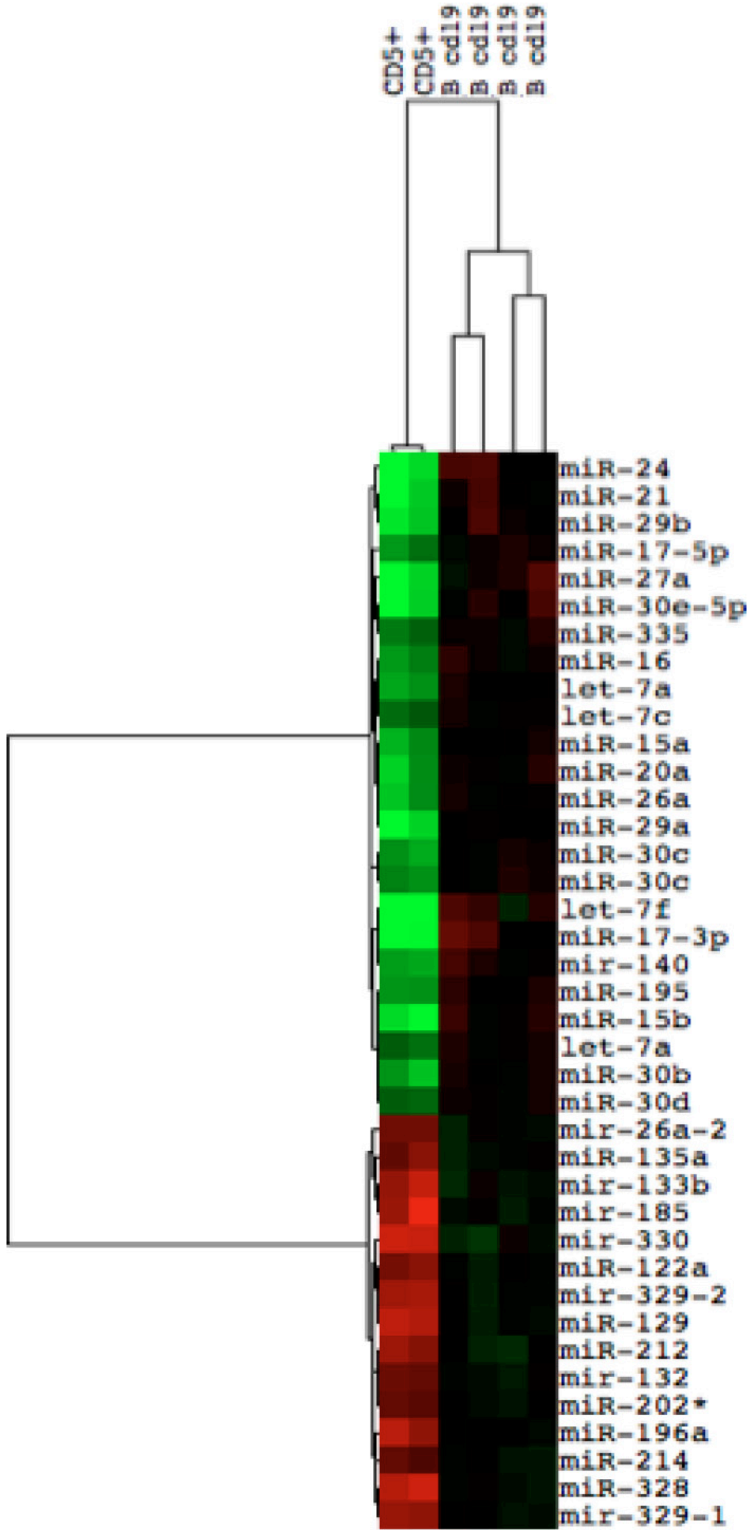

Supplementary Figure 2: Expression profile of differentially expressed miRNAs between CD19$^+$B cells from blood and CD5$^+$ B cells from tonsils. Heat map representing the level of differentially expressed miRNAs between four samples of CD19$^+$ B cells and two samples of CD5$^+$ B cells. Red: higher expression (log2, +4); green: lower expression (log2, −4).

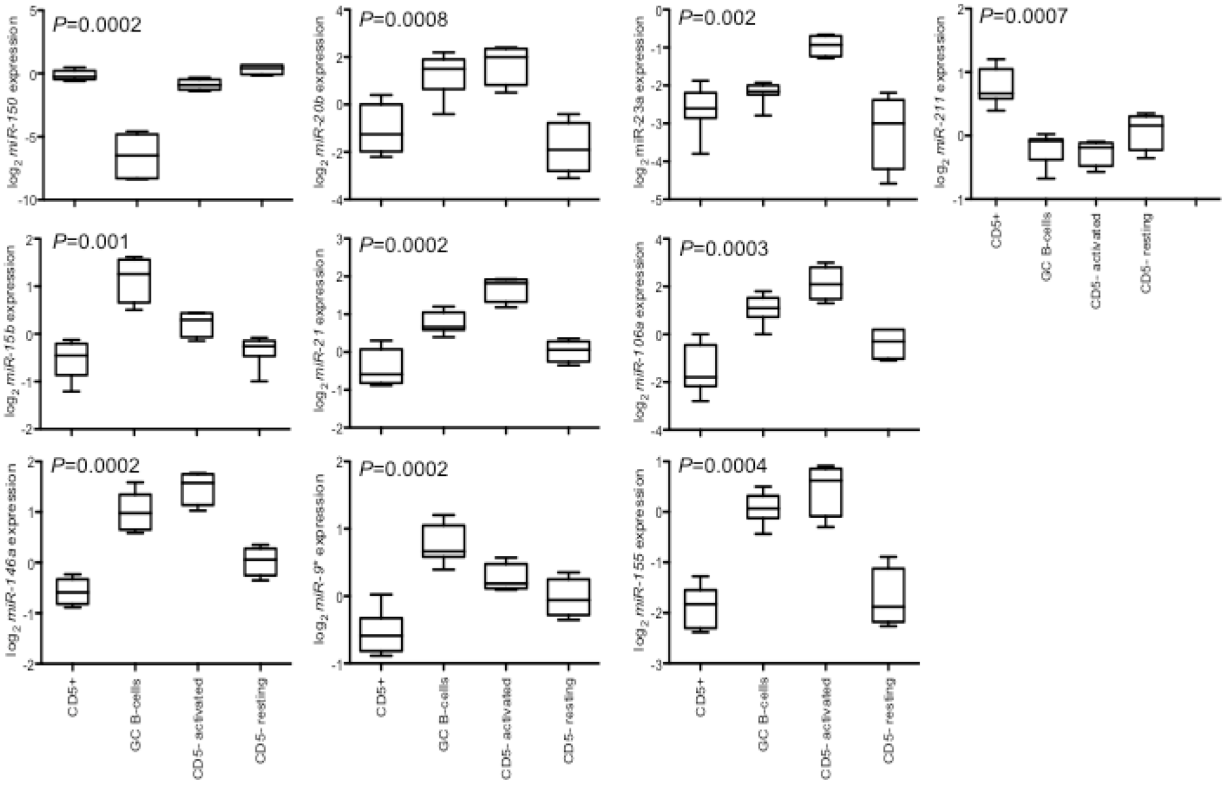

Supplementary Figure 3: Validation of the differential expression of miRNAs in B cell samples by qRT-PCR. Analysis of expression of *mir-150, mir-20b, mir-23a, mir-211, mir-15b, mir-21, mir-106a, mir-146a, mir-9** and *mir-155* in naïve $CD5^+$, GC B cells and $CD5^-$ activated and resting B cells by qRT-PCR. Box plots represent the average expression and the 25% and the 75% of data distribution. Whiskers indicate the 5% and 95% of data distribution. The data are obtained using the same B cell samples hybridized on the microarrays. In particular, 6 GC B cells and 6 $CD5^+$ B cells samples, three for each of the two pools hybridized on the array, were used. Statistical analysis of data was performed by Kruskall-Wallis test.

Supplementary Table 1: List of differentially expressed miRNAs in $CD19^+$ B cells from peripheral blood and naïve $CD5^+$ B cells, germinal centre $CD23^-$/$CD39^-$ B cells and subepithelial mature $CD5^-$ B cells from tonsils (FDR 1%). See Supplementary_Table_1

Supplementary Table 2: List of differentially expressed miRNAs between CD19⁺ B cells and naïve CD5⁺ B cells (FDR 1%)

miRNA	CD5⁺vs CD19⁺*	*Q* value
mir-329-1	Up	0.00534
mir-129	Up	0.00534
mir-328	Up	0.00534
mir-329-2	Up	0.00534
mir-30c	Down	0.00534
let-7a	Down	0.00534
mir-196a	Up	0.00545
*mir-202**	Up	0.00545
mir-15b	Down	0.00659
mir-30d	Down	0.00659
mir-122a	Up	0.00659
mir-15a	Down	0.00659
mir-132	Up	0.00659
mir-195	Down	0.00659
mir-30b	Down	0.00659
let-7c	Down	0.00694
mir-330	Up	0.00778
mir-26a-2	Up	0.00818
mir-26a	Down	0.00818
mir-212	Up	0.00818
let-7f	Down	0.00818
mir-30e-5p	Down	0.00818
mir-214	Up	0.00818
mir-16	Down	0.00818
mir-21	Down	0.00818
mir-133b	Up	0.00818
mir-29b	Down	0.00818
mir-17-5p	Down	0.00818
mir-29a	Down	0.00818
mir-335	Down	0.00818
mir-140	Up	0.00838
mir-27a	Down	0.00838
mir-17-3p	Down	0.00838
mir-24	Down	0.00838
mir-185	Up	0.00903
mir-20a	Down	0.00974
mir-135a	Up	0.00974

*Up:higher expression in naïve CD5⁺ B cells.
Down: higher expression in CD19⁺ B cells.

Supplementary Table 3: Experimentally validated target genes (according to miRTarBase, strong experimental evidences. See Supplementary_Table_3

Supplementary Table 4: Panther hallmarks significantly overrepresented among first interactors of experimentally validated target genes of differentially expressed miRNAs in B cells. See Supplementary_Table_4

Supplementary Table 5: Panther pathways significantly overrepresented among first interactors of experimentally validated target genes of differentially expressed miRNAs in B cells. See Supplementary_Table_5
