# Supplementary material for: Identification of microRNAs implicated in the late differentiation stages of normal B cells suggests a central role for miRNA targets ZEB1 and TP53
Source: Oncotarget. 2017 Jan 17;8(7):11809–26. doi: 10.18632/oncotarget.14683 (PMC5355306; doi:10.18632/oncotarget.14683)
Supplement: Supplementary file 2 [file oncotarget-08-11809-s002.docx]

**Supplementary Table 1**. List of differentially expressed miRNAs in CD19^+^ B cells from peripheral blood and naïve CD5^+^ B cells, germinal centre CD23^-^/CD39^-^ B cells and subepithelial mature CD5^-^ B cells from tonsils (FDR 1%).

| **miRNA** | **Q value** |
| --- | --- |
| *miR-150* | 9.4x10^-09^ |
| *miR-29b* | 7.6x10^-07^ |
| *miR-20a* | 1.3x10^-06^ |
| *miR-106a* | 3.2x10^-06^ |
| *miR-17-5p* | 3.2x10^-06^ |
| *miR-29a* | 4.4x10^-06^ |
| *miR-20b* | 1.4x10^-05^ |
| *miR-26a* | 1.5x10^-05^ |
| *miR-106a* | 1.5x10^-05^ |
| *miR-221* | 2.7x10^-05^ |
| *miR-93* | 2.7x10^-05^ |
| *let-7c* | 3.2x10^-05^ |
| *miR-191* | 3.7x10^-05^ |
| *miR-181b* | 4.6x10^-05^ |
| *miR-29c* | 5.0x10^-05^ |
| *miR-15b* | 5.3x10^-05^ |
| *mir-491* | 6.0x10^-05^ |
| *miR-107* | 6.0x10^-05^ |
| *miR-155* | 6.9x10^-05^ |
| *miR-24* | 8.2x10^-05^ |
| *miR-361* | 8.5x10^-05^ |
| *miR-130b* | 8.6x10^-05^ |
| *miR-181b* | 9.1x10^-05^ |
| *miR-106b* | 0.00011 |
| *mir-342* | 0.00011 |
| *miR-98* | 0.00013 |
| *let-7d* | 0.00018 |
| *mir-140* | 0.00019 |
| *miR-30a-3p* | 0.00022 |
| *miR-26a* | 0.00024 |
| *miR-138* | 0.00025 |
| *miR-29b* | 0.00025 |
| *mir-340* | 0.00026 |
| *miR-18a* | 0.00026 |
| *miR-346* | 0.00028 |
| *mir-185* | 0.00033 |
| *miR-9* | 0.00033 |
| *miR-328* | 0.00033 |
| *miR-133a* | 0.00033 |
| *mir-133b* | 0.00033 |
| *miR-129* | 0.00041 |
| *miR-103* | 0.00041 |
| *mir-30b* | 0.00042 |
| *miR-24* | 0.00042 |
| *mir-184* | 0.00043 |
| *miR-324-5p* | 0.00055 |
| *miR-196a* | 0.00055 |
| *let-7a* | 0.00057 |
| *miR-383* | 0.00062 |
| *miR-107* | 0.00062 |
| *miR-30c* | 0.00063 |
| *miR-141* | 0.00078 |
| *miR-29b* | 0.00080 |
| *miR-146b* | 0.00087 |
| *miR-219* | 0.00088 |
| *miR-146a* | 0.00091 |
| *miR-21* | 0.00093 |
| *miR-128a* | 0.00096 |
| *miR-324-5p* | 0.00104 |
| *mir-335* | 0.00104 |
| *miR-23a* | 0.00106 |
| *miR-181c* | 0.00106 |
| *miR-122a* | 0.00107 |
| *miR-135a* | 0.00119 |
| *mir-15b* | 0.00123 |
| *miR-126** | 0.00123 |
| *miR-16* | 0.00124 |
| *mir-181a-2* | 0.00126 |
| *miR-16* | 0.00126 |
| *miR-27a* | 0.00131 |
| *miR-7* | 0.00140 |
| *miR-142-5p* | 0.00148 |
| *miR-138* | 0.00150 |
| *miR-16* | 0.00150 |
| *mir-10b* | 0.00150 |
| *miR-21* | 0.00150 |
| *miR-374* | 0.00156 |
| *miR-25* | 0.00163 |
| *miR-30c* | 0.00172 |
| *let-7a* | 0.00188 |
| *miR-19b* | 0.00210 |
| *miR-30b* | 0.00213 |
| *miR-153* | 0.00213 |
| *miR-30e-5p* | 0.00221 |
| *miR-16* | 0.00230 |
| *miR-135a* | 0.00246 |
| *let-7f* | 0.00261 |
| *mir-422a* | 0.00261 |
| *miR-181a* | 0.00266 |
| *miR-518a* | 0.00284 |
| *let-7f* | 0.00284 |
| *let-7g* | 0.00305 |
| *miR-105* | 0.00309 |
| *miR-181d* | 0.00328 |
| *miR-425-5p* | 0.00331 |
| *miR-206* | 0.00331 |
| *mir-204* | 0.00331 |
| *miR-450* | 0.00333 |
| *miR-136* | 0.00342 |
| *mir-130b* | 0.00343 |
| *miR-377* | 0.00356 |
| *miR-181b* | 0.00359 |
| *mir-320* | 0.00370 |
| *miR-15a* | 0.00387 |
| *miR-183* | 0.00401 |
| *mir-330* | 0.00454 |
| *miR-181a* | 0.00488 |
| *mir-504* | 0.00488 |
| *miR-224* | 0.00493 |
| *miR-9** | 0.00493 |
| *mir-329-2* | 0.00498 |
| *miR-23b* | 0.00503 |
| *miR-184* | 0.00507 |
| *miR-92* | 0.00533 |
| *miR-17-3p* | 0.00539 |
| *miR-509* | 0.00559 |
| *miR-26a* | 0.00598 |
| *miR-15a* | 0.00712 |
| *mir-16-2* | 0.00712 |
| *mir-514-3* | 0.00770 |
| *miR-30d* | 0.00810 |
| *let-7i* | 0.00810 |
| *miR-194* | 0.00812 |
| *mir-326* | 0.00848 |
| *miR-19b* | 0.00857 |
| *miR-342* | 0.00905 |
| *miR-329* | 0.00914 |
| *miR-338* | 0.00914 |
| *miR-26b* | 0.00954 |
| *miR-320* | 0.00966 |
| *miR-195* | 0.00970 |
| *miR-222* | 0.00973 |
| *mir-206* | 0.00982 |
| *mir-193a* | 0.00982 |

**Supplementary Table 3.** Experimentally validated target genes (according to miRTarBase, strong experimental evidences. Release 4.5, Nov 1, 2014) of differentially espressed miRNAs in naïve, germinal centre and mature B cells, as reported in Table 1.

| **miRNA symbol*** | **Experimentally validated gene targets** |
| --- | --- |
| *miR-150*  *(has-mir-150-5p)* | *MYB, EGR2, VEGFA, P2RX7, IGF2, MUC4, CXCR4, ZEB1, FLT3, NOTCH3, EP300, PTPRR, MS4A3, CCNE1, AGA, ATP13A3, TP53* |
| *miR-17-5p*  *(miR-17-5p)* | *PTEN, CDKN1A, PKD2, BCL2L11, E2F1, RUNX1, MAPK9, TGFBR2, MUC17, BMPR2, CCND1, NCOA3, THBS1, RND3, SMURF1, ZNFX1, CCL1, GPR137B, NABP1, NPAT, YES1, JAK1, PTPRO, MAP3K12, BCL2, MEF2D, APP, VEGFA, DNAJC27, FBXO31, TNFSF12, MYC, SMAD4, ICAM1, SELE, CCND2, E2F3, RB1, RBL1, RBL2, WEE1, TCF3* |
| *miR-106a*  *(mir-106a-5p)* | *E2F1, FAS, CDKN1A, HIPK3, MYLIP, RB1, APP, RUNX1, ARID4B, VEGFA, IL10, TGFBR2, CYP19A1, SIRPA* |
| *miR-20a*  *(miR-20a-5p)* | *HIF1A, CCND1, E2F1, BMPR2, CDKN1A, EGLN3, TGFBR2, MAP3K12, BCL2, MEF2D, PTEN, APP, RUNX1, VEGFA, CCND2, E2F3, MAPK9, RB1, RBL1, RBL2, WEE1, KIT, IRF2, PPARG, BAMBI, CRIM1, MAP2K3, PURA, ARHGAP12, TSG101, SIRPA, TCEAL1, NRAS, BCL2L11, MUC17, MYC, BNIP2, THBS1* |
| *miR-181b*  *(miR-181b-5p)* | *NLK, GATA6, CDX2, CBX7, TIMP3, PLAG1, SIRT1, GRIA2, VSNL1, BCL2, KAT2B, TCL1A, CYLD, RNF2, TMED7, XIAP, MCL1, IGF1R, EGF1, MAP3K10* |
| *miR-361*  *(miR-361-5p)* | *VEGFA* |
| *miR-130b*  *(miR-130b-3p)* | *TP53INP1, UVRAG, PPARG, CSF1, ZEB1, DICER1, RUNX3,* |
| *miR-20b*  *(miR-20b-5p)* | *CDKN1A, EPHB4, HIPK3, MYLIP, PPARG, CRIM1, ARID4B, BAMBI, ESR1, VEGFA, EFNB2, STAT3, HIF1A, MUC17* |
| *miR-18a*  *(miR-18a-5p)* | *KRAS, ASR1, PTEN, CTGF, TNFSF11, NR3C1, TGFBR2, SMAD4, HSF2, ATM, DICER1, NCOA3, NEDD9, CDK19, SMAD3, TSC22D3* |
| *miR-29b*  *(miR-29b-3p)* | *HDAC4, CTNNBIP1, COL5A3, COL1A1, SP1, CDK6, BACE1, PPP1R13B, SFPQ, DNAJB11, NASP, DNMT3B, DNMT3A, TGFB1, TGFB2, MCL1, BCL2, DNMT1, S100B, VEGFA, TET1, TCL1A, CDC42, MMP15, MMP24, GRN, FGG, FGA, FGB, COL3A1, COL4A1, MMP2, ADAM12, NID1, BMP1, HMGA2, GSK3B, NKIRAS2, RAX, TBX21, IFNG, DUSP2, IMPDH1, MYCN, COL4A2, ACVR2A, PTEN, ESR1, NCOA3, PIK3CG, FOS* |
| *miR-148a*  *(miR-148a-3p)* | *DNMT1, HLA-G, TGIF2, DNMT3B, NR1I2, RPS6KA5, CCKBR, IRS1, ACVR1, BCL2, TMED7, CDC25B* |
| *miR-191*  *(miR-191-5p)* | *MDM4, NDST1, TMC7, IL1A, SOX4, CDK6, SATB1* |
| *miR-93*  *(miR-93-5p)* | *TP53INP1, CDKN1A, E2F1, MAPK9, VEGFA, ITGB8, KAT2B, TUSC2, PTEN, PURA, LATS2* |
| *miR-26a*  *(miR-26a-5p)* | *HMGA2, HMGA1, CCNE2, CCND2, ESR1, CDK6, PTEN, EZH2, PLAG1, SERBP1, SMAD1, RB1, MAP3K2, CCNE1, SMAD4, IFNB1, GSK3B, CPEB2, CPEB3, CPEB4, GDAP1, MTDH, ABCA1, ARL4C, IL6, CDK8, CDC6, LIF, MYC, CTGF, STRADB, E2F7, NOS2* |
| *miR-221*  *(miR-221-3p)* | *CDKN1B, BMF, FOXO3, DICER1, KIT, CDKN1C, TMED7, ETS1, HMGXB4, BBC3, ARIH2, USP18, BRAP, CREBZF, DKK2, MYBL1, TBK1, BNIP3L, DDIT4, TIMP3, DIRAS3, ICAM1, FOS, BNIP3, ESR1, TICAM1, PTEN, SELE, TRPS1, CERS2, FMR1, ZEB2, BCL2L11, HOXB5, TNFSF10, NAIP, TP53, CORO1A, TCEAL1, DVL2, POU3F2, HOXC10, MEOX2* |
| *miR-155*  *(miR-155-5p)* | *UQCRFS1, MEIS1, TAB2, MECP2, SOCS1, MSH6, MSH2, MLH1, INPP5D, DET1, SMAD5, HIVEP2, ZNF652, ZIC3, BACH1, JARID2, CSNK1A1, APC, TRIP13, TBCA, SMAD1, SDCBP, RHEB, POLE3, PKN2, PICALM, PHC2, NARS, MYO10, DHX40, CEBPB, ARID2, ARFIP1, TM6SF1, MATR3, LDOC1, PHF17, RHOA, AGTR1, PKIA, RNF123, TP53INP1, IKBKE, FGF7, KDM3A, NFATC2IP, SPI1, EDN1, FOXO3, TSHZ3, RUNX2, JUN, IFNGR1, KBTBD2, KRAS, ETS1, TLE4, CYR61, ICAM1, SELE, IRAK3, SMAD2, MYB, SKI, GCSAM, CKAP5, SOX6, CSF1R, IL13RA1, FADD, BCL6, MITF, MAP3K10, NOS3, ANAPC16, GCFC2, EXOSC2, LNX2, ZNF248, CHD9, MEF2A, CAB39, CLUAP1, CARD11, PCDH9, ZNF561, CARHSP1, LIN7C, CBR4, GPM6B, LRIF1, TAF5L, HERC4, MORC3, MBNL3, UPF2, TSPAN14, INTS6, YWHAZ, PRKAR1A, SSX2IP, FAM199X, RAC1, PLS1, SAP30L, MRPS27, CEP41, CIAPIN1, CCDC82, ACTR2, TRAK1, CYP2U1, SLC35F2, ZNF493, HAL, IL17RB, TBC1D14, ZNF254, GABARAPL1, IGJ, RAPGEF2, WBP1L, PBRM1, MRPL18, MAP3K14, ARMC2, LCORL, APAF1, MPP5, RAB11FIP2, NOVA1, RBAK, ARL15, MYO1D, LRRC59, TTF1, FAM91A1, CCDC41, KIAA0430, CDC40, DCUN1D2, KLHL5, AGO4, HBP1, WWC1, WEE1, GOLT1B, PALD1, ZNF83, PHF14, TBC1D8B, INPP5F, ARPC3, KRCC1, FAM177A1, UBTD2, SECISBP2, PAK2, SLC33A1, ZNF28, MCM8, SMARCA4, TCF12, TOMM20, UBQLN1, VPS18, WHSC1L1, MASTL, MYBL1, GATM, E2F2, FAM135A, C3orf18, ARL6IP5, PDLIM5, MSI2, DNAJC19, BRPF3, CUX1, FLI1, MYD88, OLR1, SMAD4, CD68, FLT1, VCAM1, SMAD3, IL8, ZNF611* |
| *mir-15b*  *(mir-15b-5p)* | *CCNE1, RECK, BCL2, VEGFA, EIF4A1, AXIN2, IFNG, PURA, CCND1* |
| *miR-29a*  *(miR-29a-3p)* | *BCL7A, TNFAIP3, DICER1, CDK6, CDC42, RAN, BACE1, PXDN, PPP1R13B, SERPINB9, SPARC, MYCN, DNMT3A, DNMT3B, COL4A1, COL4A2, MCL1, BCL2, CD276, DKK1, NAV3, SFRP2, ITIH5, S100B, IMPDH1, GLUL, PPM1D, PIK3R1, KREMEN2, FGG, FGA, FGB, LPL, CPEB3, CPEB4, ADAMTS9, ITGA11, NASP, SAPCD2, PTEN, ABL1, HBP1, TET1, ZFP36, DAG1, DIABLO, RET, TRIM63, TGFB3, , C1QTNF6* |
| *miR-138*  *(miR-138-5p)* | *PTK2, ARHGEF3, ROCK2, RHOC, H2AFX, SLC45A3, TERT, EID1, IGF1R, CCND3, SIRT1, FOSL1, HIF1A, CASP3, BLCAP, MXD1, RELN, EZH2, SOX4, ZEB2, VIM, GNAI2, CEBPA, PPARG, FABP4, LPL, MMP3, CDH2, PLEK2, CDH1, SNAI2, SERPINE1, EED, SUZ12* |
| *miR-106b*  *(miR-106b-5p)* | *ITHC, APP, CDKN1A, EOMES, E2F1, KAT2B, VEGFA, RB1, TCEAL1, CCND1, CCND2, E2F3, MAPK9, PTEN, RBL1, RBL2, WEE1, PURA, APC, PKD2* |
| *miR-135a*  *(miR-135a-5p)* | *JAK2, NR3C2, APC, HOXA10, MYC* |
| *miR-21*  *(miR-21-5p)* | *RASGRP1, SIRT1, EZH2, HOXA9, TP53I11, PIM1, HOXA1, FGFRL1, RAD52, CDC25A, BCL2, TM9SF3, RTN4, PLOD3, NCAPG, DERL1, BASP1, JAG1, REST, SMARCA4, RB1, ROBO1, XBP1, PTEN, FHIT, SPRY2, POU3F2, KRAS, DUSP10, TIMP3, SOX5, MTAP, DOCK7, DOCK5, RECK, PIAS3, LAMB3, TGFBR2, E2F1, TGFBI, LRRFIP1, MARCKS, SP1, GPC3, NPTX1, CCL20, TPM1, EFNA3, NFIB, CASP8AP2, APAF1, BTG2, RICTOR, PDCD4, PTPN1, BDNF, XIST, CPEB2, GPD1L, ISCU, NCAM1, E2F3, DDAH1, BIRC5, RHOB, ANP32A, ACVR2B, NR4A2, SERPINB5, LASP1, BMPR2, MECP2, RASA1, EZH2, IKBKB, SP1, NCOA3, TJP1, CAMK2G, PEA15, VOPP1, MNT, PCBP1, TRAPPC2P1, BIRC6, ACTN1, STAM2, SIRT1, CDKN1B, JMY, TOPORS, HNRNP, DAXX, TP53BP2, TP63, TGFBR3, PPIF, MAP2K3, MAPK8, MSH2, MSH6, POU4F2, TIAM1, WNK1, PPM1D, ISCU, MEF2C, PLXNB1, EIF4A2, ANKRD46, EGFR, SRGAP1, KCNMA1, IL1B, ICAM1, PLAT, PTX3, TNFAIP3, CCR1, CDC42, CD44, CDK2AP1, AIFM3, DOCK4, PTCH1, CDH5, PPARA, KRAS, ROBO1, GJA1, ING4, QKI, VMP1, TWIST1, SOST, SFRP2, NTF3, GALNT7, COL4A1, TFRC, HOXB3, TP53, FASLG, SOD3, IL11, CTNNB1, KCNJ2, DKK2, TOB1, TCF21, CDK6, SMAD7, ATF4, CSNK2A1, BMI1, BCL2L2, CTNNBIP1, SRGAP2, BCL6, PSMD10, ASF1B, TCF12, ELOVL6, TP53, WIBG, WFS1, RPS7, PDHA2, SPRY1, WNT1, RAB22A, CREB5, MBNL2, EFNA1, NUP93, MRPS27, EBP, E2F2, FMOD, CDK6, RP2, SGK3, SLC16A10, GLCCI1, SOCS5, SESN1, PRRG4, HIPK3, FAM3C, FAS, CDKN1A, P4HB, SMCHD1, TNPO1, CBX1, ABCB9, CDK10, DENND6A, HOXA3, KIAA1161, MDGA1, MID1IP1, SEH1L, UBQLN1, SERTAD2, ACVR1B, APC, ATP11C, CHD9, CLASP2, ELK3, PTAR1, NIPBL, MIB1, HECTD1, NFE2L1, MAFG, MRE11A XPA, TGIF1, MYC, ERBB2, NACC1, DAPK1, SMAD7, CCNA2, CCNB1, VEGFA, MMP2, MMP9, ELAVL4, AKT2, STAT3* |
| *mir-16-2*  *(mir-16-2-3p)* | *RARB* |
| *miR-181c*  *(miR-181c-5p)* | *NLK, GATA6, CDX2, NOTCH4, KRAS, NOTCH2, BCL2, TRIM2, SIRT1, BTBD3, BMPR2, IL2* |
| *miR-141*  *(miR-141-3p)* | *MAPK14, ZEB2, ZEB1, DLX5, BAP1, ACVR2B, KLF5, STK3, CDYL, CTBP2, TGFB2, SFPQ, CLOCK, BRD3, UBAP1, PTEN, ZFPM2, TRAPPC2P1, EIF4E, PPARA, NROB2, YWHAG, SHC1, VAC14, TCF7L1, ELMO2, RASSF2, KLHL20, RIN2, SEPT7, HOXB5, ERBB2IP, KLF11, WDR37, ELAVL4, MAPK9, TFDP2, E2F3* |
| *mir-185*  *(mir-185-5p)* | *RHOA, CDC42, SIX1, NTRK2, DNMT1, EPAS1, AR, SCARB1, CCNE1, CDK6, AKT1, HMGA2, CORO2B* |
| *miR-149*  *(miR-149-5p)* | *AKT1, E2F1, SP1, MYBL2, FOXM1* |
| *miR-181d*  *(miR-181d-5p)* | *BCL2, HRAS, MGMT* |
| *miR-425-5p*  *(miR-425-5p)* | *CCND1, PTEN, THRB, FGFR3, TACC3, MAFB* |
| *miR-29c*  *(miR-29c-3p)* | *IGFBP1, TFAP2C, COL21A1, COL3A1, COL1A1, CDC42, TDG, SPARC, LAMC1, COL15A1, COL4A2, COL4A1, COL1A2, GAPDH, PPP1R13B, DNMT3B, CDK6, MCL1, BCL2, FBN1, SRSF10, FGG, FGB, FGA, WNT4, MYCN, BACE1* |
| *miR-181a*  *(miR-181a-5p)* | *DUSP6, NLK, GATA6, CDX2, PRAP1, PLAG1, RALA, PTPN22, PTPN11, DUSP5, RNF2, BCL2, PROX1, KAT2B, CDKN1B, ZNF763, DDIT4, ATM, HIPK2, BCL2L11, HRAS, SIRT1, FOS, MTMR3, KLF6, MCL1, XIAP, GPR78, NANOG, NOTCH1* |
| *miR-25*  *(miR-25-3p)* | *BCL2L11, CCL26, CDKN1C, KAT2B, TP53, CDH1, WDR4, MDM2, PTEN, EZH2, SMAD7, PRMT5, KLF4,* |
| *mir-204*  *(mir-204-5p)* | *MEIS1, BCL2, FOXC1, TGFBR2, SNAI2, TGFBR1, MEIS2, SNAI1, SPDEF, THRB, CDX2, AP1S2, BCL2L2, BIRC2, EDEM1, EZR, FZD1, IL11, M6PR, RAB22A, RAB40B, SERINC3, SERP1, TCF12, TCF4, RUNX2, SOX4, EFNB2, ALPL, SOST, CREB5, HOXA10, MCL1, MAP1LC3B, ELOVL6* |
| *mir-184*  *(mir-184)* | *AKT2, INPPL1, NFATC2* |
| *miR-148b*  *(miR-148b-3p)* | *HLA-G, CCKBR, ITAG5, ROCK1, PIK3CA, NRAS, CSF1* |
| *miR-373*  *(miR-373-3p)* | *RAD23B, RECK, VEGFA, LATS2, SIRT1, CD44, RASSF1, MBD2, RAD52, TXNIP, RABEP1, MTOR, NFIB, CSDC2, XPA, MRE11A* |
| *miR-9**  *(miR-9-3p)* | *RCOR11, ITGFB1* |
| *mir-30b*  *(mir-30b-5p)* | *SOCS1, BCL6, SMAD1, CCNE2, SNAI1, CAT* |
| *miR-19b*  *(miR-19b-3p)* | *BACE1, PTEN, ATXN1, HIPK3, ARID4B, MYLIP, ESR1, KAT2B, SOCS1, BCL2L11, BMPR2, TLR2, PRKAA1, PPPR5E, CYP19A1, GCM1, MYCN, NCOA3, TGFBR2, CUL5* |
| *mir-320*  *(mir-320a)* | *AQP4, AQP1, NPR1, TAC1, TFRC, MCL1, HSPB6, MAPK1, POLR3D* |
| *miR-146a*  *(miR-146a-5p)* | *CXCR4, TLR2, FADD, TRAF6, IRAK1, ROCK1, BRCA2, BRCA1, TLR4, FAF1, NFKB1, CDKN1A, EGFR, CD40LG, FAS, ERBB4, SMAD4, WASF2, L1CAM, CFH, IRAK2, CCNA2, PA2G4, SNAP25, IL8, MTA2, CDKN3, KIF22, NFIX, BGLAP, SPP1, SLP1* |
| *miR-30a-3p*  *(miR-30a-3p)* | *CDK6, SLC7A6, TMEM2, THBS1, CYR61, VEZT, TUBA1A, KRT7, WDR82* |
| *miR-9*  *(miR-9-5p)* | *MMP13, REST, CDH1, POU2F2, BCL6, ETS1, RAB34, BACE1, PRDM1, FOXO1, NFKB1, NTRK3, NR2E1, ONECUT2, CDX2, AP3B1, CCNG1, SRF, SIRT1, TGFBI, SOCS5, ID2, FOXO3, CCND1, STMN1, GRN, VIM, CHMP2B, CD34* |
| *mir-323*  *(mir-323-3p)* | unknown |
| *mir-211*  *(mir-211-5p)* | *KCNMA1, CDH5, POU3F2, CREB5, ELOVL6, TCF12, RAB22A, IL11* |
| *miR-328*  *(miR-328)* | unknown |
| *mir-335*  *(mir-335-5p)* | *MAPK1, TNC, MERTK, PTPRN2, SOX4, TFF2, BCL2L2, LRG1, RASA1, ARPC5L, RB1, UBE2F, RUNX2, BIRC5, SP1, ID4, IGF1R, EPN2, MYC, BRCA1* |
| *miR-15a*  *(miR-15a-5p)* | *BMI1, WNT3A, MYB, CDC25A, CCND2, BCL2, CCND1, CCNE1, BACE1, MN1, CRKL, CLCN3, DMTF1, BRCA1, AKT3, FGF7, CADM1, TMEM184B, APP, UCP2, VEGFA, TSPYL2, CHUK, TP53, HMGA1, HMGA2, IFNG, PURA, RECK, PDCD4, RAB21, WT1, SKAP2, NFKB1, DLK1* |

* in parentheses the official miRNA symbol from www. mirbase.org

| **Supplementary Table 4.** Panther hallmarks significantly overrepresented among first interactors of experimentally validated target genes of differentially expressed miRNAs in B cells | | | |  |
| --- | --- | --- | --- | --- |
| **Description** | **Gene reference list** | **Genes in Overlap** | **FDR**  **Q-value** |  |
| Genes up-regulated during transplant rejection. | 200 | 70 | 7.09x10^-57^ |  |
| Genes up-regulated by activation of the PI3K/AKT/mTOR pathway. | 105 | 48 | 7.47x10^-46^ |  |
| Genes involved in the G2/M checkpoint. as in progression through the cell division cycle. | 200 | 61 | 7.91x10^-46^ |  |
| Genes important for mitotic spindle assembly. | 200 | 59 | 1.66x10^-43^ |  |
| A subcluster of genes regulated by MYC - version 1 (v1). | 200 | 58 | 2.14x10^-42^ |  |
| Genes encoding cell cycle related targets of E2F transcription factors. | 200 | 52 | 1.85x10^-35^ |  |
| Genes mediating programmed cell death (apoptosis) by activation of caspases. | 161 | 44 | 3.82x10^-31^ |  |
| Genes regulated by NF-kB in response to TNF [GeneID=7124]. | 200 | 48 | 3.87x10^-31^ |  |
| Genes involved in p53 pathways and networks. | 200 | 46 | 4.87x10^-29^ |  |
| Genes up-regulated in response to IFNG [GeneID=3458]. | 200 | 45 | 5.00x10^-28^ |  |
| Genes encoding components of apical junction complex. | 200 | 42 | 5.65x10^-25^ |  |
| Genes up-regulated by IL6 [GeneID=3569] via STAT3 [GeneID=6774]. e.g.. during acute phase response. | 87 | 29 | 1.08x10^-23^ |  |
| Genes down-regulated in response to ultraviolet (UV) radiation. | 144 | 32 | 4.54x10^-20^ |  |
| Genes encoding components of the complement system which is part of the innate immune system. | 200 | 35 | 2.29x10^-18^ |  |
| Genes defining inflammatory response. | 200 | 35 | 2.29x10^-18^ |  |
| Genes up-regulated by STAT5 in response to IL2 stimulation. | 200 | 34 | 1.73x10^-17^ |  |
| Genes up-regulated by activation of WNT signaling through accumulation of beta catenin CTNNB1 [GeneID=1499]. | 42 | 18 | 1.84x10^-17^ |  |
| Genes up-regulated through activation of mTORC1 complex. | 200 | 33 | 1.19x10^-16^ |  |
| Genes up-regulated in response to TGFB1 [GeneID=7040]. | 54 | 18 | 3.36x10^-15^ |  |
| Genes up-regulated in response to ultraviolet (UV) radiation. | 158 | 28 | 3.82x10^-15^ |  |
| Genes defining early response to estrogen. | 200 | 30 | 3.82x10^-14^ |  |
| Genes up-regulated during unfolded protein response. a cellular stress response related to the endoplasmic reticulum. | 113 | 23 | 5.19x10^-14^ |  |
| Genes defining late response to estrogen. | 200 | 27 | 9.18x10^-12^ |  |
| Genes defining response to androgens. | 101 | 19 | 3.69x10-^11^ |  |
| Genes involved in development of skeletal muscle (myogenesis). | 200 | 25 | 2.81x10^-10^ |  |
| Genes up-regulated in response to low oxygen levels (hypoxia). | 200 | 24 | 1.46x10^-9^ |  |
| Genes involved in DNA repair. | 150 | 20 | 5.72x10^-9^ |  |
| Genes up-regulated by activation of Notch signaling. | 32 | x10 | 1.04x10^-8^ |  |
| Genes encoding components of peroxisome. | 104 | 16 | 2.49x10^-8^ |  |
| Genes up-regulated during production of male gametes (sperm). as in spermatogenesis. | 135 | 18 | 3.09x10^-8^ |  |
| Genes up-regulated by KRAS activation. | 200 | 22 | 3.09x10^-8^ |  |
| Genes up-regulated in response to alpha interferon proteins. | 97 | 15 | 5.92x10^-8^ |  |
| Genes defining epithelial-mesenchymal transition. as in wound healing. fibrosis and metastasis. | 200 | 21 | 1.32x10^-7^ |  |
| Genes involved in metabolism of heme (a cofactor consisting of iron and porphyrin) and erythroblast differentiation. | 200 | 21 | 1.32x10^-7^ |  |
| Genes encoding proteins over-represented on the apical surface of epithelial cells. e.g.. important for cell polarity (apical area). | 44 | 10 | 2.41x10^-7^ |  |
| Genes encoding proteins involved in glycolysis and gluconeogenesis. | 200 | 20 | 5.52x10^-7^ |  |
| A subgroup of genes regulated by MYC - version 2 (v2). | 58 | 10 | 3.43x10^-6^ |  |
| Genes down-regulated by KRAS activation. | 200 | 17 | 3.28x10^-5^ |  |
| Genes up-regulated during formation of blood vessels (angiogenesis). | 36 | 7 | 4.59x10^-5^ |  |
| Genes up-regulated by activation of hedgehog signaling. | 36 | 7 | 4.59x10^-5^ |  |
| Genes encoding components of blood coagulation system; also up-regulated in platelets. | 138 | 13 | 9.39x10^-5^ |  |
| Genes up-regulated during adipocyte differentiation (adipogenesis). | 200 | 16 | 1.02x10^-4^ |  |
| Genes encoding proteins involved in processing of drugs and other xenobiotics. | 200 | 16 | 1.02x10^-4^ |  |
| Genes involved in protein secretion pathway. | 96 | 10 | 2.54x10^-4^ |  |
| Genes encoding proteins involved in oxidative phosphorylation. | 200 | 15 | 3.25x10^-4^ |  |
| Genes specifically up-regulated in pancreatic beta cells. | 40 | 6 | 6.36x10^-4^ |  |
| Genes involved in cholesterol homeostasis. | 74 | 6 | 1.42x10^-2^ |  |
| Genes involve in metabolism of bile acids and salts. | 112 | 7 | 3.00x10^-2^ |  |
| Genes up-regulated by reactive oxigen species (ROS). | 49 | 4 | 4.05x10^-2^ |  |

| **Supplementary Table 5**. Panther pathways significantly overrepresented among first interactors of experimentally validated target genes of differentially expressed miRNAs in B cells | | | |
| --- | --- | --- | --- |
| **Description** | **Gene**  **reference list** | **Genes in overlap** | **FDR**  **Q-value** |
| CCKR signaling map | 169 | 102 | 1.66x10^-64^ |
| Gonadotropin releasing hormone receptor pathway | 225 | 89 | 1.44x10^-41^ |
| Angiogenesis | 154 | 73 | 7.15x10^-39^ |
| EGF receptor signaling pathway | 130 | 65 | 7.92x10^-36^ |
| Apoptosis signaling pathway | 115 | 61 | 5.86x10^-35^ |
| Interleukin signaling pathway | 97 | 57 | 6.91x10^-35^ |
| B cell activation | 65 | 47 | 1.73x10^-32^ |
| T cell activation | 80 | 49 | 1.14x10^-30^ |
| Inflammation mediated by chemokine and cytokine signaling pathway | 245 | 75 | 8.44x10^-28^ |
| Integrin signalling pathway | 181 | 65 | 1.04x10^-27^ |
| PDGF signaling pathway | 138 | 56 | 2.78x10^-26^ |
| Parkinson disease | 107 | 49 | 3.93x10^-25^ |
| Ras Pathway | 79 | 43 | 8.01x10^-25^ |
| Wnt signaling pathway | 309 | 79 | 2.21x10^-24^ |
| p53 pathway | 81 | 41 | 1.78x10^-22^ |
| FGF signaling pathway | 123 | 48 | 1.05x10^-21^ |
| VEGF signaling pathway | 59 | 35 | 3.03x10^-21^ |
| DNA replication | 29 | 25 | 1.03x10^-18^ |
| Alzheimer disease-amyloid secretase pathway | 65 | 32 | 4.87x10^-17^ |
| Toll receptor signaling pathway | 56 | 29 | 6.00x10^-16^ |
| Interferon-gamma signaling pathway | 32 | 23 | 1.80x10^-15^ |
| p53 pathway feedback loops 2 | 51 | 27 | 5.04x10^-15^ |
| TGF-beta signaling pathway | 94 | 35 | 5.18x10^-15^ |
| p38 MAPK pathway | 39 | 23 | 1.16x10^-13^ |
| Insulin/IGF pathway-protein kinase B signaling cascade | 39 | 23 | 1.16x10^-13^ |
| Huntington disease | 163 | 42 | 1.16x10^-12^ |
| PI3 kinase pathway | 49 | 23 | 1.27x10^-11^ |
| Oxidative stress response | 27 | 18 | 2.07x10^-11^ |
| JAK/STAT signaling pathway | 18 | 15 | 9.86x10^-11^ |
| Alzheimer disease-presenilin pathway | 122 | 32 | 1.15x10^-09^ |
| Endothelin signaling pathway | 87 | 27 | 1.25x10^-09^ |
| FAS signaling pathway | 34 | 17 | 8.18x10^-09^ |
| Cadherin signaling pathway | 170 | 35 | 7.67x10^-08^ |
| Insulin/IGF pathway-mitogen activated protein kinase kinase/MAP kinase cascade | 33 | 15 | 3.88x10^-07^ |
| Hypoxia response via HIF activation | 28 | 13 | 3.46x10^-06^ |
| Cytoskeletal regulation by Rho GTPase | 95 | 21 | 6.99x10^-05^ |
| p53 pathway by glucose deprivation | 24 | 10 | 3.69x10^-04^ |
| Muscarinic acetylcholine receptor 1 and 3 signaling pathway | 50 | 14 | 4.10x10^-04^ |
| 5HT2 type receptor mediated signaling pathway | 51 | 14 | 5.13x10^-04^ |
| Nicotine pharmacodynamics pathway | 33 | 11 | 9.61x10^-04^ |
| Heterotrimeric G-protein signaling pathway-Gq alpha and Go alpha mediated pathway | 113 | 21 | 9.98x10^-04^ |
| Axon guidance mediated by netrin | 34 | 11 | 1.27x10^-03^ |
| Hedgehog signaling pathway | 22 | 9 | 1.35x10^-03^ |
| Histamine H1 receptor mediated signaling pathway | 29 | 10 | 1.89x10^-03^ |
| Oxytocin receptor mediated signaling pathway | 44 | 12 | 2.68x10^-03^ |
| Thyrotropin-releasing hormone receptor signaling pathway | 46 | 12 | 4.11x10^-03^ |
| Ubiquitin proteasome pathway | 63 | 14 | 5.19x10^-03^ |
| Axon guidance mediated by semaphorins | 22 | 8 | 9.58x10^-03^ |
| Transcription regulation by bZIP transcription factor | 51 | 12 | 1.09x10^-02^ |
| P53 pathway feedback loops 1 | 8 | 5 | 2.01x10^-02^ |
| Angiotensin II-stimulated signaling through G proteins and beta-arrestin | 39 | 10 | 2.16x10^-02^ |
| Dopamine receptor mediated signaling pathway | 57 | 12 | 3.01x10^-02^ |
